# Converging Evidence Supporting the Cognitive Link between Exercise and Esport Performance: A Dual Systematic Review

**DOI:** 10.3390/brainsci10110859

**Published:** 2020-11-15

**Authors:** Adam J. Toth, Niall Ramsbottom, Magdalena Kowal, Mark J. Campbell

**Affiliations:** 1 The Department of Physical Education and Sport Science, University of Limerick, V94 T9PX Limerick, Ireland; adam.toth@ul.ie (A.J.T.); niall.ramsbottom@ul.ie (N.R.); magdalena.kowal@ul.ie (M.K.); 2 Lero, the Science Foundation Ireland Research Centre for Software, University of Limerick, V94 NYD3 Limerick, Ireland

**Keywords:** exercise, cognition, video gaming, esports, cognitive psychology, neurocognition, Physical exercise

## Abstract

(1) Background: Research into action video games (AVG) has surged with the popularity of esports over the past three decades. Specifically, evidence is mounting regarding the importance of enhanced cognitive abilities for successful esports performance. However, due to the sedentary nature in which AVGs are played, concerns are growing with the increased engagement young adults have with AVGs. While evidence exists supporting the benefits of exercise for cognition generally in older adult, children and clinical populations, little to no work has synthesized the existing knowledge regarding the effect of exercise specifically on the cognitive abilities required for optimal esports performance in young adults. (2) Method: We conducted a dual-systematic review to identify the cognitive abilities integral to esports performance (Phase 1) and the efficacy of exercise to enhance said cognitive abilities (Phase 2). (3) Results: We demonstrate the importance of four specific cognitive abilities for AVG play (attention, task-switching, information processing, and memory abilities) and the effect that different types and durations of physical exercise has on each. (4) Conclusion: Together, these results highlight the role that exercise can have on not only combating the sedentary nature of gaming, but also its potential role in facilitating the cognitive aspects of gaming performance.

## 1. Introduction

Video game play has surged in popularity over the last 30 years, becoming a ubiquitous part of modern culture. Recent reports estimate that 91% of children play video games in the United States alone and that the number of worldwide video gamers will rise to over 2.7 billion by 2021 [1]. Moreover, gaming industry revenue now exceeds that of the film and music industries combined [2,3,4]. This rise in video game popularity can be traced to the advent of improved and readily available broadband as well as organised competitive video game play, otherwise known as esports [5]. This growing interest in esports has led to new research seeking to understand both the performance, as well as the health benefits and pitfalls associated with competitive video gaming (e.g., [6,7]).

Currently, video games are utilised in numerous research contexts, including the study of emotion (for a review, see [8]), motivation (e.g., [9]), skill learning (e.g., [10]), and rehabilitation of neurological disorder (e.g., [11]). This research, in addition to new work dispelling the myth that action video games (AVGs) overtly influence aggressive behaviour [12,13], has demonstrated a notable shift towards examining the positive impact of video gaming on numerous populations. One area gaining significant research attention is the effect of action video games (AVGs) on cognition [14]. The AVG game genre, which includes first-person shooters (FPS), third-person shooters (TPS) and Multiplayer Online Battle Arena (MOBA) games, is currently responsible for the largest and most popular esports in the world. Such esports include Counterstrike: Global Offensive (CSGO), Defense of the Ancients (DOTA) 2 and League of Legends (LoL), which has a total of over 140 million monthly players [15,16,17].

### 1.1. Gaming and Cognition

Research examining the link between AVG play and cognitive ability has predominantly focused on either comparing the cognitive performance of AVG players (AVGPs) to non-gamer (NGs) controls or investigating the change in cognitive performance following an AVG intervention. For example, AVGPs are shown to display superior information processing ability compared to NGs [18]. Additionally, AVG play has shown to improve attentional allocation, spatial processing, and mental rotation abilities (for a review, see [19]). In fact, the magnitude of AVG-mediated spatial skill improvement is shown to compare to the improvements observed following engagement with formal courses (secondary school and university) aimed at enhancing those same skills [3,20].

Despite mounting evidence in favour of the association between AVG play and improved cognition, some question the efficacy of video games for improving cognitive abilities. Specifically, [21] concluded in their meta-analysis that ‘playing video games has negligible effects on cognitive ability’ (p. 3). However, their broad search criteria focused primarily on non-action video games (e.g., Tetris and The Sims), even though such games have been shown to less readily affect cognition compared to AVGs [14]. Overall, there is consensus that due to their fast pace and complexity, AVGs place a unique demand on a number of cognitive abilities, including attentional control, working memory and executive function [18,22].This demand appears to enhance the performance of AVGPs on a number of validated cognitive ability tests when compared to NGs and in so doing, highlights AVGPs as a desirable research population in the field of cognitive neuroscience.

### 1.2. Exercise and Cognition

The importance of cognitive ability for competitive gaming performance has led to the exploration of ways in which esport teams can improve the cognitive abilities of their players to gain an advantage. One strategy that has not been studied is the implementation of physical exercise in the routines of gamers [23]. It has been suggested that the recent attention towards physical exercise in esports has largely been due to the apparent sedentary nature of playing video games that has been linked to muscular-skeletal disorders and obesity issues [24,25]. However, despite remaining seated for multiple hours, [26] have found that elite gaming can in fact create high physical demand, evidenced by high heart rates and cortisol levels measured during competition [27]. Given these demands and how frequently esport athletes practice, professional teams have employed personal trainers to design physical exercise programmes to maintain the physical health of players (e.g., the esport franchise; Team Liquid; [23,28]).

While exercise may assist players with managing the physical demands of esports, the role that exercise can have on the cognitive abilities required for esports performance has received little attention to date. Physical exercise has been suggested to lead to improvements in attention [29], long-term memory [30], learning [31], and motor skill acquisition abilities [32], even after acute exercise [29,33,34]. Exercise has also been linked to improving neural efficiency in the prefrontal cortex and has been demonstrated to promote neurogenesis and brain plasticity [35]. However, the cognitive benefit of exercise remains debated with one point of contention being the lack of a clear description of exercise interventions employed in cognition research [36].

Exercise can be characterised based on a number of criteria, including whether it is aerobic or anaerobic, dynamic or static, blocked or varied. It is important to consider these characteristics as evidence suggests that cognition is differentially affected by various exercise features. For instance, Ref. [37] demonstrated that acute aerobic exercise can significantly improve reaction time during a working memory task, whereas no such effects were observed after resistance exercise. Additionally, Ref. [38] reported low intensity exercise led to a significant increase in resting state functional connectivity (rs-FC) in the left and right Frontoparietal Network (FPN), while rs-FC decreased in the Somatosensory Network and the Dorsal Attention Network after high intensity exercise. These results suggest that transient persistent network alterations after exercise can be differentially moderated by varying exercise intensity. Such studies exemplify how exercise type and intensity can influence exercise’s effect on cognitive ability and how this information may be significant for developing tailored physical exercise programmes for cognitively driven sports [5].

Finally, while physical exercise has been shown to enhance cognitive ability in individuals with and without mental disorders [39,40], in older adults [41], and in children [42], debate remains on the effect of exercise on the cognitive abilities of young healthy adults. While early studies failed to find any effect of exercise on young adults’ visual search task [43] and letter detection task performance [44], more recent work has shown cognition to benefit from exercise. However, debate continues over the benefits following various types of exercise and whether cognitive benefits are domain specific [45]. To the best of our knowledge, very little work to date has specifically examined the effects of physical exercise on cognition in young healthy adults, e.g., [46]. Given that over 50% of worldwide gamers are between the ages of 20 to 40 [1], with professional esports players peaking during their early to mid-twenties [47], identifying the cognitive skills affected by AVGs and the physical exercises that may enhance those same abilities in young adults will add to our understanding about the role of physical exercise on the cognitive and gaming performance among esports athletes and habitual action video gamers.

The purpose of the current review is three-fold. First, it aims to synthesize the existing literature both examining the effect of gaming as an intervention on cognition and comparing the cognitive abilities between AVGPs and NGs. By doing so, we will identify the cognitive abilities demanded by action video games for successful performance. Secondly, this review aims to synthesize the existing literature examining the effect of different exercise interventions on cognitive ability. Finally, we will converge the evidence from this dual-systematic review to identify the exercise characteristics that mediate exercise-induced improvements in the cognitive abilities specifically identified to be pertinent for action video game play.

## 2. Phase 1 Methods

**Figure 1:**
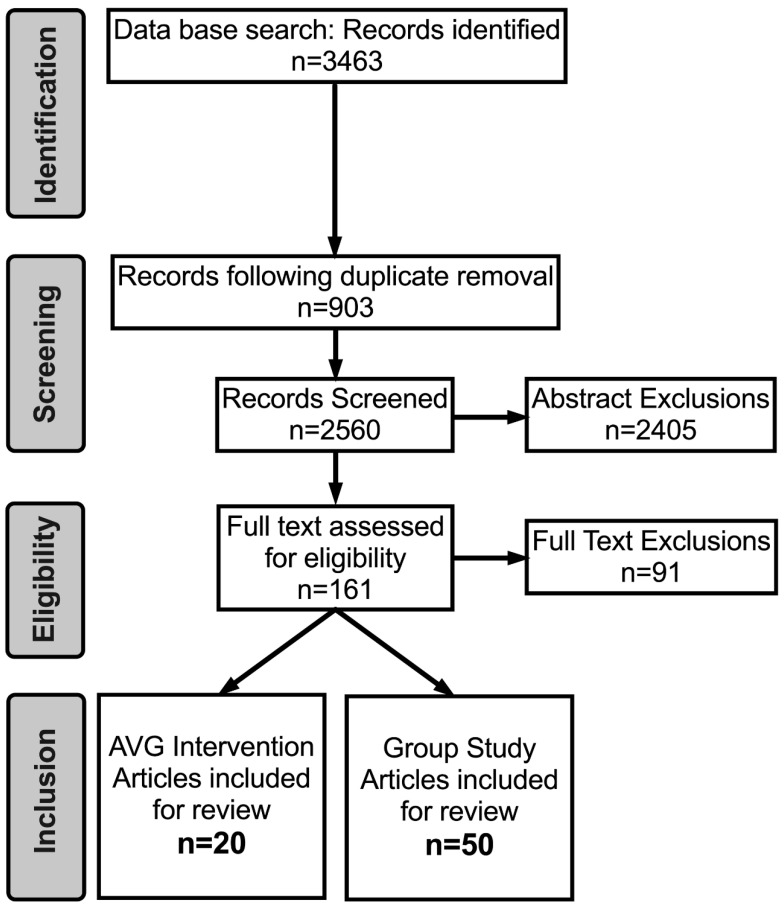
Flow chart demonstrating the identification, screening, eligibility and inclusion of articles identified for phase 1 (gaming and cognition) of the dual systematic review in accordance with PRISMA guidelines.

This systematic review was conducted according to the PRISMA guidelines [48] (see PRISMA Guidelines checklist in Supplementary Materials File S1). The search for Phase 1 of this review was conducted by NR. Literature searches were first performed using Pub Med, PsycINFO, Google scholar, and MEDLINE online databases to identify research articles that assessed the effect of an AVG intervention on cognitive ability between 1999 and 30 July 2019 (when the search was conducted). The year 1999 was chosen as it is considered to signify the emergence and mainstream recognition of esport [49]. Articles were retrieved if combinations of keywords associated with video gaming AND cognition were found in the title or abstract of each article (see Supplementary Materials File S2 for the procedure and syntax used for each database). Identified articles were extracted and exported into Endnote (Clarivate Analytics). NR screened each of the identified articles based on the inclusion criteria and AT and MC were involved in the collective decision regarding each article’s inclusion. Reference lists from retrieved papers were screened for further relevant articles (Figure 1).

### 2.1. Inclusion Criteria

In total, 3463 article abstracts were screened and excluded based on the following inclusion criteria.

(1) Participants tested were young healthy adults between the ages of 18 and 45.
(2) For intervention studies, the intervention must be an action video game (AVG). Bediou and colleagues [50] definition of AVGs (first-person shooter games, wherein the player views the world through the eyes of his or her avatar, and third-person shooter games, wherein the player sees the back of his or her avatar) was used as a guide with the additional inclusion of the Multiplayer Online Battle Arena (MOBA) genre of games. We also required that AVGs be commercially available and not designed for the purpose of research (e.g., Space Fortress).
(3) Intervention studies must be conducted using a pre-post design with an age-matched control group.
(4) For group studies, a clearly defined group of action video game players (AVGPs) must be compared to an age-matched non-gamer (NG) control group.
(5) The study must have directly assessed the impact of a gaming intervention or compared the performance between AVGPs and NGs on a specific cognitive ability.

Based on these criteria, 3302 studies were excluded from further analyses (Figure 1).

### 2.2. Selection of Studies

The remaining 161 articles were read and summarized based on the AVG intervention used and cognitive ability assessed. The most common reason for exclusion at this stage was that studies failed to use an AVG intervention or they failed to use a pre-post design.

### 2.3. Quality Assessment

The 11 item PEDro scale [51] was completed to assess the methodological quality of the experimental research and to assess risk of bias for each individual study, thus, allowing classification of high- or low-quality scientific studies based on cut offs (see Supplementary Materials File S3). Items 6 and 9 of the PEDro scale were considered not relevant to the current review as they directly assessed criteria related to physiotherapy research practices. Overall, studies were considered excellent in quality by scoring 7–9, good quality by scoring 4–6, and fair quality by scoring 3–4. Of the 20 intervention studies included in this review, all were scored as ‘excellent quality’ due to the strict inclusion criteria.

### 2.4. Quantifying the Effect of AVG Studies on Cognitive Ability

In order to estimate the potential efficacy of an AVG intervention on improving cognitive ability, we divided the number of studies examining a particular cognitive ability that showed a positive effect by the total number of included studies, and multiplied the result by 100 (see Equation (1)).

(1) $$Success\;Rate=\;\left(\frac{Studies\;with\;Positive\;Effect_{Cognitive\;Ability}}{Total\;Studies_{Cognitive\;Ability}}\right)\times 100$$

This percentage provided an observed estimate of the success rate for an AVG intervention to improve a given cognitive ability, or, in the case of group studies, the success rate for a group study to demonstrate superior cognitive performance of AVGPs compared to NGs. However, the reliability of this success rate estimate can fluctuate based on the number of studies included in its calculation. In order to assess the confidence that a calculated success rate represents the true success rate, we also calculated the probability that the observed success rate represented the true success rate given the included studies. This metric allows for a more informed comparison of the estimated success rates among AVG intervention (or group) studies across the cognitive abilities, where the number of studies contributing to the success rate for each cognitive ability differ. We used a Bayesian approach to calculate this probability, *p*(*A*|*Data*), according to Equation (2), where *p*(*A*) represents the prior probability that the true success rate is a given value, *p*(*Data*|*A*) is the probability of observing the number of positive effects out of the number of total studies given the true success rate, and *p*(*Data*) is the probability of observing the number positive effects out of the total number of studies [52].

(2) p(A|Data)=p(A)p(Data|A)/p(Data)

We assumed having no prior information about the true success rate and therefore, assigned all true success rates from 1% to 100% a uniform prior probability of 0.01 (i.e., *p*(*A*) = 0.01). *p*(*Data*|*A*) was calculated from the binomial distribution (Equation (3)), where *n* represents the total number of studies, *k* represents the number of studies demonstrating AVG play to improve the cognitive ability examined, and *A* represents the true success rate (on a scale of 0–1) of the AVG intervention on the cognitive ability.

(3) $$p\left(Data|A\right)=\left(\begin{array}{c} n\\ k \end{array}\right)A^{k}{\left(1-A\right)}^{n-k}$$

By calculating *p*(*Data*|*A*) for every possible *A* from 0.1 to 1, we created a binomial distribution for the effect of an AVG intervention on each cognitive ability. This distribution was multiplied by the prior distribution (see Equation (2)) and then finally normalized by dividing by *p*(*Data*), which was calculated as the integrated area underneath the binomial distribution curve. The resulting probability density functions demonstrate the probability that, given the data, the true success rate lies between any two values between 0 and 1.

Lastly, we used Laplace’s rule of succession ([53] Laplace, 1814/1951) to estimate the probability that a future additional AVG intervention or group study would find a positive association between AVG play and cognition or show superior cognitive performance of AVGPs compared to NGs, given the included studies analysed, where *n* represents the number of currently included studies, and *k* represents the number of positive effects among those included studies (Equation (4)).

(4) $$p\left(Positive\;Effect_{n+1}\right)=k+1/n+2$$

## 3. Results

### 3.1. Study Characteristics

Overall, 70 studies fully met the inclusion criteria (Figure 1) and were divided into two sub-categories: intervention studies (*n* = 20; those that examined the effect of an AVG intervention on a specific cognitive ability compared to a non-gaming control) and group studies (*n* = 50; those that compared the cognitive abilities between AVGPs and NGs). Details for each intervention and group study can be found in Table 1 and Table 2, respectively.

### 3.2. Intervention Studies

The 20 intervention studies identified for inclusion in this review included a total of 798 participants (44% female) ranging from 17 to 45 years of age (Mean age = 22.76) and were published between 2003 and 2017. A variety of AVG interventions, such as Medal of Honor, Halo, Call of Duty, and Unreal Tournament franchises, were used to assess attention, memory, information processing and task-switching cognitive abilities, with intervention durations varying between a single 25-min session to 50 h across multiple weeks (see Table 1).

#### 3.2.1. Attention

**Figure 2:**
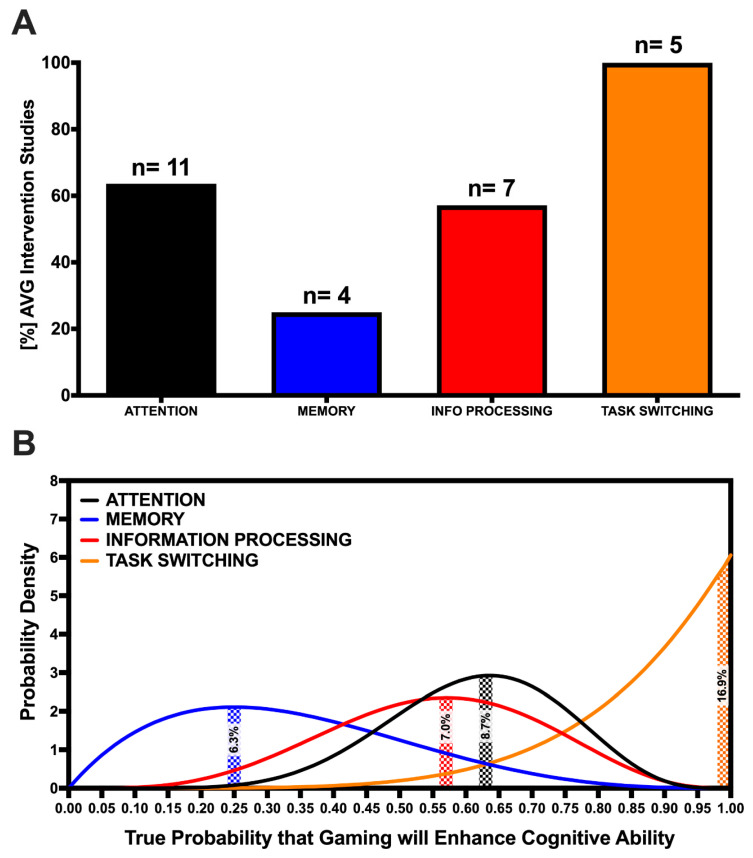
(**A**) Percent of included studies finding a positive effect of AVG play on attention, memory, information processing and task-switching cognitive abilities. ‘*n*’ values represent the total number of included studies examining each cognitive ability. (**B**) Probability density functions for the true success rates of a study to find attention (*black trace*), memory (*blue*), information processing and task-switching (orange) performance improvements following AVG play, respectively. Shaded areas represent the probability (/100%) that the observed success rate, given the findings of the included studies, reflects the true success rate.

Eleven intervention studies examined the effect of AVG play on the attentional ability of 470 participants (Mean = 42.73, SD = 21.46), who had a mean age of 23.12 (SD = 1.91). All of these studies used numerous previously validated visual search tasks to assess visual attention, with the Useful Field of View (UFOV) test most commonly used [14,55,56]. The average AVG intervention duration was 11.14 h (SD = 7.34) and varied from a single 25-min session to 15 sessions across 4 weeks. Seven of the 11 studies found a positive effect of the intervention on visual attention performance, resulting in an observed success rate of 63.63% (Figure 2A). Given the number of included studies, the probability that 63.63% is the true success rate of an AVG intervention on attention is 8.7% (Figure 2B). According to Laplace’s rule of succession, the probability of finding a new AVG intervention study to show attentional ability improvement following AVG play, given the included results, is 61.54%.

#### 3.2.2. Memory

Four intervention studies examined the effect of AVG play on memory and focused largely on working memory and short-term memory. Studies included 238 participants (Mean = 59.50, SD = 19.69) (Mean Age = 22.33, SD = 2.32). One study showed a positive effect of AVG play on memory, resulting in a 25% observed success rate and 6.3% probability of the observed rate being the true success rate (Figure 2). Boot and colleagues [55] employed a battery of tests, including the Visual Short-Term Memory test [71], the Corsi block-tapping task [72] and the Spatial 2-back [73], to assess short term and spatial memory. Similarly, Blacker and colleagues [63] assessed visual working memory capacity and precision using the Change Detection task and Colour Wheel task, respectively. Sanchez [60] used paper folding and card rotation tests to assess working memory and [62] used the Theory of Visual Attention (TVA) task, which also assess visual short-term memory storage capacity. The average AVG intervention was 16.36 h (SD = 12.31) and varied from a single session to 15 sessions across 4 weeks. Ref. [65] found a significant effect of AVG play on memory performance following their 30-h training intervention across 30 days, whereas [55,60,62] failed to find any positive effects from their interventions (Table 1). The probability of a new study finding AVG play to improve memory given the included results based on Laplace’s rule of succession is 40%.

#### 3.2.3. Information Processing

Seven studies including 275 participants (Mean = 39.29, SD = 20.64) with a mean age of 21.90 (SD = 1.83) examined the effect of AVG play on information processing ability. These studies used the mental rotation task [55,56], emotion search task [22], enumeration task [65]), lateral masking paradigm, and a moving dots task [66], among others to assess information processing ability. The average AVG intervention duration among these studies was 20.14 h (SD = 16.13), varying from a single session to 50 sessions across 9 weeks. Four of the seven studies found AVG play to significantly improve information processing ability, an observed success rate of 57.14% (Figure 2A). The confidence that this observed success rate represented the true success rate that an AVG intervention would improve information processing ability, given the number of included studies, is 7.0% (Figure 2B). Interventions ranging from 10 to 50 h were found to all improve information processing performance [55,56,65,66]. The probability that a new study would find AVG play to improve information processing given the current results here according to Laplace’s rule of succession is 55.56%.

#### 3.2.4. Task-Switching

Five intervention studies included in this review examined the effect of AVG play on task-switching abilities in 181 participants (Mean = 36.20, SD = 12.69) with a mean age of 23.39 (SD = 2.16). Interventions included the AVGs Call of Duty, Medal of Honor, and Unreal Tournament franchises, and the average intervention lasted 31 h (SD = 18.84) across multiple weeks. Each study found AVG play to significantly enhance task-switching ability, an observed success rate of 100% with at 16.9% probability of this being the true success rate given the number of included studies (Figure 2). The switch-cost paradigm [19], Multiple object tracking task [65], Visual and dual search task [70], Dual-task and task-switching paradigms [69], and the Multi-Attribute Task Battery (MATB; [68]) were among those tests used to assess task-switching performance. According to Laplace’s rule of succession, the probability of a future study finding AVG play to improve task-switching ability given the current results is 85.71%.

### 3.3. Group Studies

The 50 group studies included in this review directly compared the cognitive abilities of habitual AVGPs to NGs. The group studies reviewed included 3740 healthy adult participants (Mean = 74.80, SD = 124.72) and evaluated the same cognitive abilities identified in the above intervention studies (See Table 2).

**Table 2:** Study characteristics for studies comparing the cognitive performance of action video game players (AVGPs) and non gamers (NGs). Articles are organised by the cognitive ability investigated.

Cognitive Ability	Authors (Year)	Sample Size	Age: Mean (±SD)	Cognitive Test(s)	Result
Visual Attention	Antzaka et al. (2017) [74]	40	20.83 (± 2.73)	Global Report; Partial Report; Challenging Reading Task	*
	Bavelier et al. (2012) [75]	26	20.50 (± NA)	Visual Search Task	*
	Castel, Pratt, and Drummond (2005) [76]	40	20.90 (± NA)	Visual Search Task	=
	Chisholm and Kingstone (2015) [77]	32	21.35 (± NA)	Compound Search Task	*
	Chisholm et al. (2010) [78]	32	21.30 (± NA)	Attentional Capture Task	*
	Chisholm and Kingstone (2012) [79]	32	21.50 (± NA)	Oculomotor Capture Task.	*
	Dye and Bavelier (2010) [80]	47	20.30 (± 1.45)	UFOV and Attentional Blink	*
	Dye, Green and Bavelier (2009) [81]	27	20.15 (± 1.34)	ANT	*
	Gorbet and Sergio (2018) [82]	20	25.20 (± 6.46)	Visuomotor Mapping	*
	Green and Bavelier (2003) [14]	16	NA (± NA)†	Enumeration, UFOV, Attentional Blink	*
	Green and Bavelier (2005) [83]	46	19.68 (± NA)	Enumeration task	*
	Green and Bavelier (2006) [65]	16	20.90 (± NA)	Target Localization Task	*
	Howard, Wilding, and Guest (2016) [84]	43	22.09 (± NA)	Rapid Serial Visual Presentation Task	*
	Hubert-Wallander et al. (2011) [85]	55	19.80 (± NA)	Two visual search tasks	*
	Krishnan et al. (2012) [86]	24	NA (± NA)†	Visual Search Task	*
	Li et al. (2016) [58]	52	22.50 (± NA)	Lane Keeping task and Visuomotor Control Task.	*
	Mack and Ilg (2014) [87]	46	19.05 (± 0.6)	2 Occulomotor Tasks	*
	Mack, Wiesmann, and Ilg (2016) [88]	98	18.00 (± 0.20)	Spatial Cueing Task	*
	Morin-Moncet et al. (2016) [89]	24	24.35 (± 3.64)	Serial Reaction Time Task	*
	Richardson and Collaer (2011) [90]	81	19.50 (± 1.80)	Judgment of Line Angle, Position and UFOV Tests	*
	Schubert et al. (2015) [62]	34	24.50 (± 3.35)	TVA	*
	Sungur and Boduroglu (2012) [91]	44	20.50 (± 1.20)	UFOV Test	*
	Unsworth et al. (2015) [92]	198	19.49 (± 1.75)	SART and Antisaccade Task	=
	Wang et al. (2017) [93]	62	28.99 (± 3.73)	Attention Cancellation Test, ANT	*
	West et al. (2015) [94]	59	24.15 (± 3.77)	4-on-8 Virtual Maze and Visual Attention ERP Task	*
	Wilms et al. (2013) [95]	42	17.50 (± 0.15)	TVA, Enumeration Test and the ANT.	=
	Wong et al. (2018) [96]	113	22.28 (± 3.12)	Attentional Blink Task	*
	Wu and Spence (2013) [70]	36	21.55 (± NA)	Visual search task and dual search task	*
Information Processing	Appelbaum et al. (2013) [97]	125	21.45 (± NA)	Partial Report Performance Task	*
	Cain et al. (2014) [98]	40	21.75 (± NA)	Anti-Cueing Task	*
	Clark et al. (2011) [99]	32	21.49 (± 3.52)	Change Detection Task	*
	Green and Bavelier (2007) [100]	20	20.00 (± NA)	Target Identification Task	*
	Kowal et al. (2018) [18]	155	21.40 (± 2.50)	TMT-A	*
	Latham et al. (2014) [101]	40	24.45 (± 1.02)	Line Bisection Task	*
	Pavan et al. (2016) [102]	24	NA (± NA)†	Random Dot Kinematogram Task	*
	Pohl et al. (2014) [103]	60	23.70 (± NA)	Prime Discrimination Task	*
	Richardson and Collaer (2011) [90]	81	19.50 (± 1.80)	Mental Rotation Test	*
	Richlan et al. (2018) [104]	28	23.04 (± 3.07)	Visuospatial Task and Letter Detection Task	=
	Steenbergen et al. (2015) [105]	36	21.80 (± 0.86)	Stop-Change Paradigm	*
	Wang et al. (2017) [93]	62	28.99 (± 3.73)	TMT-A and Rey Complex Figure task	*
	West et al. (2008) [106]	24	19.50 (± NA)	Temporal Order Judgment and Signal Detection Paradigm	*
	West et al. (2015) [94]	59	24.15 (± 3.77)	4-on-8 Virtual Maze and Visual Attention ERP task	*
Memory	Appelbaum et al. (2013) [97]	125	21.45 (± NA)	Partial Report Performance Task	=
	Blacker and Curby (2013) [107]	121	21.60 (± 3.50)	Coloured Stimuli Test and Complex Shapes Task	*
	Cardoso-Leite et al. (2016) [108]	60	20.68 (± 0.63)	AX-Continuous Performance, N-back, and Filter Tasks	*
	Sungur and Boduroglu (2012) [91]	44	20.50 (± 1.20)	Colour Wheel Task	*
	Unsworth et al. (2015) [92]	198	19.49 (± 1.75)	Operation Span, Symmetry Span and Reading Span Tasks	=
	Wang et al. (2017) [93]	62	28.99 (± 3.73)	Wordlist Recall and N-Back	*
	Wilms et al. (2013) [95]	42	17.50 (± 0.15)	A test based on the TVA, Enumeration test and ANT	*
Task-Switching	Cain et al. (2012) [109]	44	21.65 (± NA)	Congruent and Incongruent Task Switching Tasks	*
	Cardoso-Leite et al. (2016) [108]	60	20.68 (± 0.63)	Task-Switching Test	*
	Donohue et al. (2010) [110]	45	19.94 (± 2.5)	Simultaneity Judgment Task and Temporal-Order Judgment Task	*
	Gaspar et al. (2013) [111]	60	21.90 (± 2.70)	High-Fidelity Street Crossing Simulator	=
	Green and Bavelier (2006) [83]	46	19.68 (± NA)	MOT test	*
	Green et al. (2012) [19]	78	19.91 (± NA)	Task-Switching Paradigm	*
	Karle, Watter and Shedden (2010) [112]	96	18.78 (± NA)	Two Task-Switching Paradigms	*
	Kowal et al. (2018) [18]	155	21.40 (± 2.50)	TMT-B	*
	Strobach et al. (2012) [68]	20	25.10 (± 6.26)	Dual-task test and Task-Switching test	*
	Sungur and Boduroglu (2012) [91]	44	20.50 (± 1.20)	Multiple Identity Tracking Task	*
	Wang et al. (2017) [93]	62	28.99 (± 3.73)	Number Switch Task	*

* AVG players performed significantly better than non-gamers. ^=^ No difference between AVGPs and NGs. UFOV = Useful Field of View. SART = Sustained Attention Reaction Time. TVA = Theory of Visual Attention. ANT = Attentional Network Test. TMT = Trail Making Test. MOT = Multiple Object Tracking AVGPs = Action Video Game Players NGs = Non-gamers. † age data not available but reported testing undergraduate students.

#### 3.3.1. Attention

**Figure 3:**
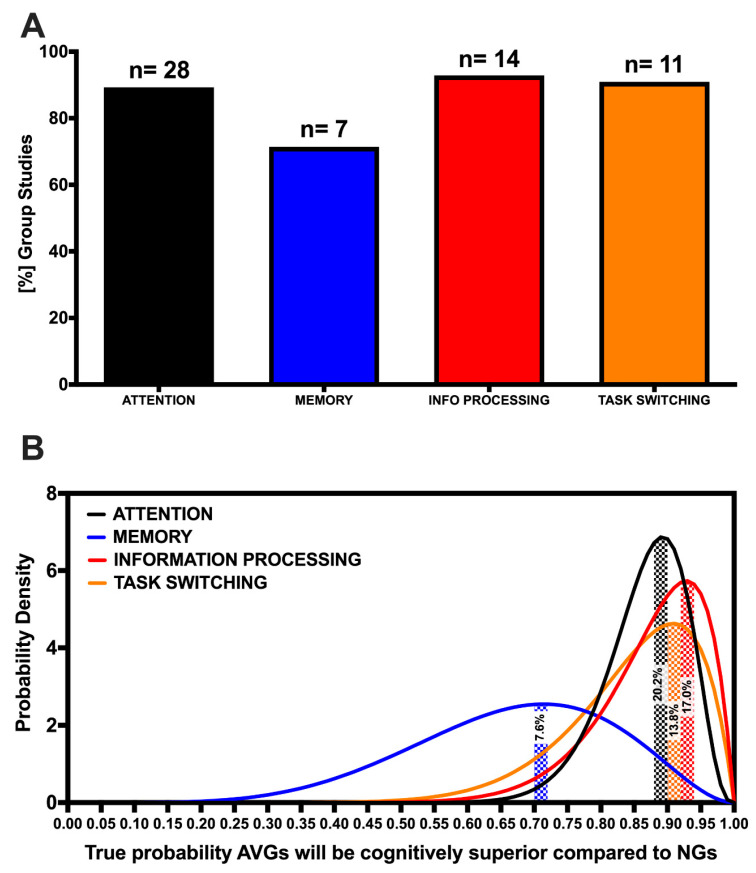
(**A**) Percent of included studies finding a AVG players (AVGPs) to outperform non-gamers (NGs) on tests of attention, memory, information processing and task-switching cognitive abilities. ‘*n*’ values represent the total number of included studies examining each cognitive ability. (**B**) Probability density functions for the true success rates of a study to find AVGPs to outperform NGs on tests of attention (*black trace*), memory (*blue*), information processing and task-switching (orange) ability, respectively. Shaded areas represent the probability (/100%) that the observed success rate, given the findings of the included studies, reflects the true success rate.

Twenty-eight of the 50 group studies compared attention ability between habitual AVGPs and a NG control sample. Cognitive tests of attention, including the Enumeration test, UFOV [113], Serial Reaction Time Task [114], and Sustained Attention Reaction Time (SART) [115] test. Those who played AVGs regularly outperformed those who did not regularly play video games in 25 of the 28 included studies (89.29% success rate) on multiple aspects of attention, such as sustained attention, reaction time, and attentional control (Figure 3A, Table 2). The probability that this observed success rate reflects the true success rate, given the studies included, is 20.2% (Figure 3B). Primarily, AVGPs outperformed NGs on tests of spatial attention [65] and reaction time [89]. Moreover, two studies utilized fMRI and EEG brain imaging techniques, respectively, and implicated the frontoparietal network in enhanced visual attention (i.e., gamers engaged this network to a lesser extent than non-gamers, thus, resulting in more efficient allocation of attention [73,86]). According to Laplace’s rule of succession, the probability of finding AVGPs to show superior attentional ability compared to NGs in a future study, based on the included results, is 86.67%.

#### 3.3.2. Memory

Seven studies employed validated memory tasks, including the N-back [108], Wordlist Recall [93], the Complex shapes task [107] and the Partial Report Performance task [97] to compare the memory ability of AVGPs and an age-matched NG control. Five studies found AVGPs to significantly outperform NGs working memory and short-term memory, equating to a 71.4% observed success rate among included studies (Figure 3A, Table 2). The probability the observed success rate reflects the true success rate for group studies to find superior memory ability in AVGPs compared to NGs, given the included studies here, is 7.6% (Figure 3B). Finally, based on the current findings, we calculated that a future study would have a 66.67% chance of finding AVGPs to show superior memory ability compared to NGs according to Laplace’s rule of succession.

#### 3.3.3. Information Processing

All but one (92.86%) of the fourteen studies comparing information processing abilities between AVGPs and NGs included in this review found AVGPs to significantly outperform NGs on tests of information processing ability (Figure 3A, Table 2). The probability that this observed success rate reflects the true success rate for AVG group studies to show superior information processing ability of AVGPs compared to NGs is 17.0% given the included studies (Figure 3B). Multiple aspects of information processing were assessed using tests such as the TMT-A [18], line bisection task [101], change detection task [99], and mental rotations test [90]). AVGPs outperformed NGs on measures of visual and spatial aspects of information processing speed. Moreover, despite failing to find differences in overall task performance, an fMRI study by Richlan and colleagues [104] identified a significant increase in the blood oxygen level dependent (BOLD) signal in frontoparietal regions from baseline to post-test, reflecting increased neural engagement in AVGPs compared to NGs. According to Laplace’s rule of succession, a future study would have a 93.75% chance of finding AVGPs to show superior information processing ability compared to NGs.

#### 3.3.4. Task-Switching

Ten out of 11 studies that examined task-switching ability between AVGPs and NGs found AVGPs displayed superior task-switching ability, equating to an observed success rate of 90.91% (Figure 3A, Table 2). The probability that this observed success rate reflects the true success rate of finding AVG group studies to show AVGPs to have superior task-switching ability compared to NGs, given the included studies, is 13.8% (Figure 3B). These studies used validated cognitive tests such as the Trail Making Test B (TMT-B) [18], Number Switch Task [93], and Multiple Identity Tracking Task [91]. Applying Laplace’s rule of succession demonstrated that a future study would have an 84.62% chance of finding AVGPs to show superior task-switching ability compared to NGs, based on the included findings.

## 4. Discussion

The first phase of this dual systematic review highlights those cognitive abilities enhanced by AVG play and superiorly shown by AVGPs when compared to NG controls. The intervention studies included in this systematic review show that AVG play may improve attention, information processing, and task-switching abilities in non-gamers, suggesting that these cognitive abilities are demanded by prominent AVGs for successful performance. While memory does not appear as likely to improve following AVG play, the current evidence is not yet convincing, as true success rates both below and above chance for the effect of AVG play to improve memory ability remain equally likely (Figure 2B). Further to this, our results also demonstrate that among studies comparing AVGP and NG performance on tests of the same cognitive abilities, AVGPs are found to predominantly outperform NGs. Taken together, while the work to date suggests the cognitive abilities outlined above appear important for successful esports performance, further research is required to increase confidence in these observed effects and to establish the causal link between improved cognitive ability and enhanced AVG performance.

### 4.1. Attention

Previous work has shown attentional ability to be important for esports performance (see [50,116]). Fast paced AVGs such as LoL and CSGO present a wide range of visual stimuli that gamers must filter and rapidly react to in order to gain an advantage over their opponents. In this review, AVG intervention studies largely focused on attention, with 7 out of 11 finding AVG play to significantly improve performance. These 7 studies used varying intervention durations from a single 25-min session [60] to 35 h across 5 weeks [61]. The fact that AVG play could enhance attention may also extend beyond the realm of esports. For example, the ability for AVG play to improve attentional ability may also have implications in clinical research areas, where bespoke video game environments may be used to facilitate the rehabilitation of individuals with attention deficit disorder, where improving attentional processing has already been shown to alleviate symptoms in this population [117,118].

While the current results suggest AVG play may improve attentional ability, the importance of future research to strengthen confidence in the true effect is required. Furthermore, a focus on optimising the moderators of the observed effect, including session duration, may show the true effect of AVG play on attention to be even greater than indicated here. Examining evidence from group studies, we similarly noticed that AVGPs display superior attentional ability compared to NGs based on performance on an array of tests. Together, the evidence from both intervention and group studies suggests that AVGs place a unique demand on attention such that attentional ability can be improved through AVG play.

### 4.2. Memory

AVGs often require players to maintain and update a plethora of information in their memory in order to gain a competitive advantage over their opponents. As such, previous work has investigated and highlighted memory to be a key cognitive ability linked to action video game play [119]. However, the majority of studies demonstrating a positive association between AVG play and memory performance compare the memory performance of AVGPs and NGs. We saw that 71.4% of the group studies in phase 1 of this review found AVGPs showed superior memory performance compared to NGs (Figure 3A). Among the few studies using AVG play as an intervention to improve memory, findings suggest that AVG play is not efficacious for improving memory. Based on this, it may be that individuals with superior memory ability are increasingly drawn toward engaging in AVG play, when compared to individuals with inferior memory ability. Alternatively, and in support of the evidence that AVGPs outperform NGs on tests of memory, it may be that the enhanced memory displayed by AVGPs develops over much longer periods of AVG engagement. For example, Blacker and colleagues [63] found significant improvements in working memory following 30 h of AVG play across 30 days. As confidence in the observed success rate for AVG intervention studies to show a positive effect of AVG play on memory is very low (based on the contribution of only four studies), with Figure 3B showing that the true success rate of an AVG intervention on improving memory could realistically lie anywhere between 0% to 75%, we encourage future research to investigate the prolonged effect of AVG play on memory in a non-gaming population to establish more clearly the efficacy of AVG play on memory ability.

### 4.3. Information Processing

Information processing speed capabilities also play a key role in esports performance as gamers are presented with rapidly changing multisensory stimuli for prolonged periods of time [120]). Therefore, those who can process information efficiently are more likely to out-perform competitors. We found four of the seven intervention studies included in this phase of our review showed participants improved information processing ability following AVG training. These studies used information processing speed tests such as the Enumeration Task and Marching Figures task. The AVG session interventions ranged from 10–50 h across multiple weeks. We also found that 13 of 14 studies showed AVGPs to outperform NGs on tests of information processing ability, providing strong evidence regarding the importance of this cognitive ability for AVG play. Our results corroborate the work of [121], who also found that video game play was associated with improved information processing in both group and intervention studies. Overall, our results demonstrate here that information processing ability may play a key role in esports performance.

### 4.4. Task-Switching

Finally, the fluid nature of AVGs requires players to constantly allocate their attention between many tasks, suggesting that superior cognitive flexibility and task-switching abilities are required cognitive skills for esports performance [19]. For example, LoL players must constantly switch focus between objectives and enemies throughout a complex map over the course of a match. Our results demonstrate that both AVG intervention and group studies are successful at showing task-switching ability improvements and differentiating the superior performance of AVGPs compared to NGs 100% and 90.91% of the time, respectively (Figure 2A and Figure 3A). Again, more than double the number of group studies met our inclusion criteria compared to AVG intervention studies, demonstrating the need for further work to increase confidence in the true efficacy of AVG play for improving task-switching ability. The importance of task-switching ability for gaming also indirectly demonstrates the importance that sleep can have on gaming performance. Recent work has highlighted that sleep loss specifically hinders the ability to update task-relevant information in response to changing circumstances [122,123]). This link between sleep and task-switching ability may be especially relevant for esports athletes, where research regarding the sleep quantity and quality of athletes has yet to be investigated and would provide valuable insight into the link between cognitive and esports performance.

### 4.5. Limitations

A key limitation among the AVG intervention studies identified throughout this review is the variance in intervention duration. Interventions varied from a single 25-min session to 50 h across 4 weeks. This variance makes it difficult to definitively conclude whether the few AVG intervention studies improve the different cognitive abilities examined in this review and highlights the need for research investigating whether a dose response exists regarding the effect of AVG interventions for improving cognitive ability. Secondly, the variation in the types of tests used to measure a single cognitive ability, and their administration, across studies highlights the lack of consensus over the most robust measurement tool for different cognitive abilities. For example, [55] used multiple tests administered to participants on three separate occasions, whereas [67] used a single administration of the Moving Dots Task. These differences may influence participants’ motivation and/or attention, and may explain performance differences across studies. Generally, research in cognitive science should look to better establish the most reliable and robust measurement tools for establishing cognitive aptitude, including the details around their administration, thus providing a clear guide for researchers as to the appropriate tools to use when investigating cognition in any population.

### 4.6. Conclusions

This first phase of our dual systematic review highlights the importance of attention, memory, information processing and task-switching abilities for esports performance. Initial evidence from intervention studies demonstrates that these cognitive abilities are linked to AVG play supporting the claim that AVGs place a high demand on these skills. These findings are more strongly supported by group studies that show habitual AVGPs outperform NGs on the same cognitive skills. Based on these findings, including our assessments using Laplace’s Rule of succession across the cognitive abilities examined in both intervention and group studies, we suggest that Task-Switching Ability, Information Processing and Attention, and Memory are the most-to-least important for action video gaming. While this proposed order of importance may change as future work is conducted to strengthen our findings and increase confidence in true success rates, especially related to the effect of AVG interventions on cognitive performance, we present this categorisation as a first guide on the relative importance of different cognitive abilities for action video game performance. In establishing the apparent importance of these cognitive abilities for gaming performance, we now look to investigate the effect of physical exercise on these specific cognitive abilities in young healthy adults in phase two of this dual systematic review, to inform how physical exercise may indirectly impact gaming performance via cognitive enhancement.

## 5. Phase 2: Methods

**Figure 4:**
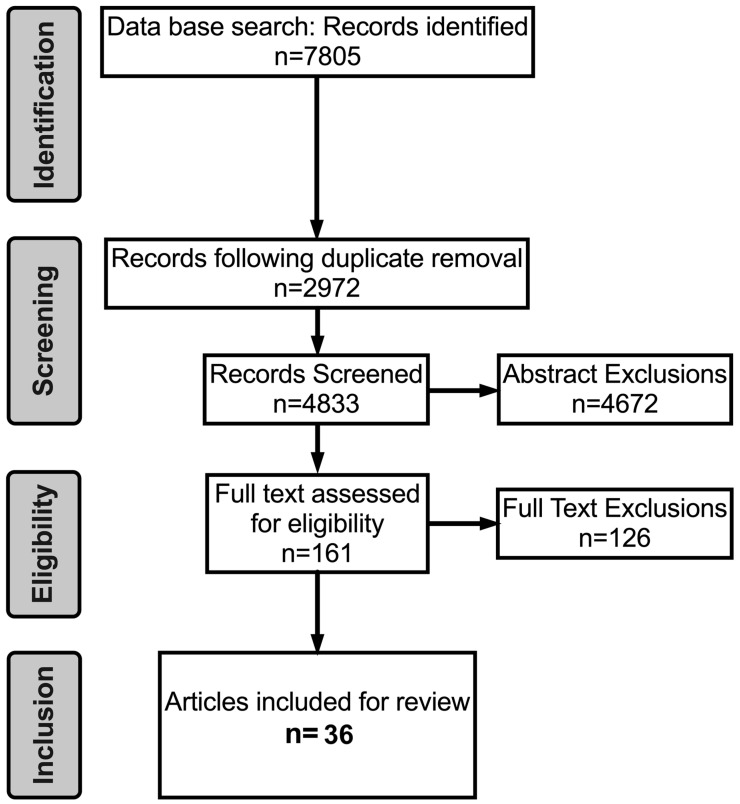
Flow chart demonstrating the identification, screening, eligibility and inclusion of articles identified for phase 2 (exercise and cognition) of the dual systematic review in accordance with PRISMA guidelines.

Phase 2 of this systematic review was conducted according to the PRISMA statement [48] (see PRISMA Guidelines checklist in Supplementary Materials File S1). The search for Phase 2 was conducted by NR and MK. MK searched PsychINFO and Medline, and NR searched PubMed and Google Scholar to identify primary research articles that assessed the effect of an exercise intervention on cognitive ability between 1999 and July 2019, i.e., the same timeframe as in Phase 1. The same filters were used on each database and all identified articles were extracted and exported into Endnote (Clarivate Analytics). Both authors were involved in the screening process and where there was any discrepancy regarding an article’s inclusion, AT and MC served as arbitrators and a collective decision was made. The search for Phase 2 was completed in November of 2019. As the cognitive abilities of attention, information processing, memory, and task-switching were shown to be either enhanced through AVG training or superiorly displayed by AVGPs compared to NGs, these results informed the search terms for Phase 2. Thus, articles were retrieved if keywords related to exercise combined with keywords related to cognitive ability were found in the title or abstract of each article. A final search in each database was run with filters that corresponded with our inclusion criteria (see Supplementary Materials File S4, for procedure and search syntax). Reference lists from retrieved papers and existing reviews were screened for further relevant articles (Figure 4).

### 5.1. Inclusion Criteria

A total of 7805 article abstracts were screened in EndNote and excluded based on the following inclusion criteria.

(1) Participants were young healthy adults between the ages of 18 and 45.
(2) The study used a pre-post design in which an exercise intervention group was compared to an age-matched control group.
(3) The control group was a non-exercise control group.
(4) The study used a reliable and validated cognitive test to assess a specific cognitive ability.
(5) The intervention group conducted exercise under normal conditions (i.e., not hypoxia or sleep deprivation).

Based on these criteria and following the removal of duplicates, 7644 studies were excluded from further analyses, leaving 161 articles (Figure 4).

### 5.2. Selection of Studies

The remaining 161 articles were read and summarised based on the type, duration and intensity (low, moderate and high) of the exercise intervention used as well as the cognitive ability assessed. The most common reason for exclusion at this stage was that pre and post cognitive measures were not recorded and/or a non-exercise control was not included. In total, 36 articles were included for quality assessment (see Figure 4). We would like to express our appreciation to anonymous reviewer 2 who identified one additional study that was not discovered initially or upon a second conducting of our search, but did meet our inclusion criteria, and was therefore subsequently included in Phase 2 of our dual-systematic review.

### 5.3. Quality Assessment

As in phase 1, a modified version of the PEDro scale [51], with items 6 and 9 omitted, was completed to assess the quality of the included scientific studies and to assess risk of bias [124]. Of the 36 studies included, all were considered to be of ‘excellent quality’ (See Supplementary Materials File S5).

### 5.4. Quantifying the Effect of Exercise on Cognitive Performance

As in phase 1, we used Equations (1)–(3) to calculate the observed success rates and probability density functions for the positive effect of exercise interventions on each cognitive ability. We also used Laplace’s rule of succession to calculate, for each cognitive ability, the probability that a new exercise intervention study would show exercise to improve cognitive ability.

## 6. Phase 2: Results

### 6.1. Study Characteristics

Overall, 36 intervention studies published between 2003 and 2019 fully met the inclusion criteria (See Figure 4) and tested 1807 (Mean = 50.19, SD = 43.32) participants ranging in age from 18–45 (Mean = 22.73, SD = 2.45). Studies were sub-divided based on the cognitive ability they assessed and then further examined based on exercise type (i.e., aerobic exercise, HIIT, resistance training) and intensity (low, moderate, high). All studies assessed the same cognitive abilities shown to be important to esports performance in phase 1 of this dual systematic review and details for each are shown in Table 3.

### 6.2. Cognitive Abilities

The effect of exercise was investigated on the following cognitive abilities: Attention (*n* = 26), Memory (*n* = 14), Information Processing (*n* = 8) and Task-switching (*n* = 4). As several studies utilised multiple cognitive tests, some contribute towards examining the effect of exercise on multiple cognitive abilities.

#### 6.2.1. The Effect of Exercise on Attention

**Figure 5:**
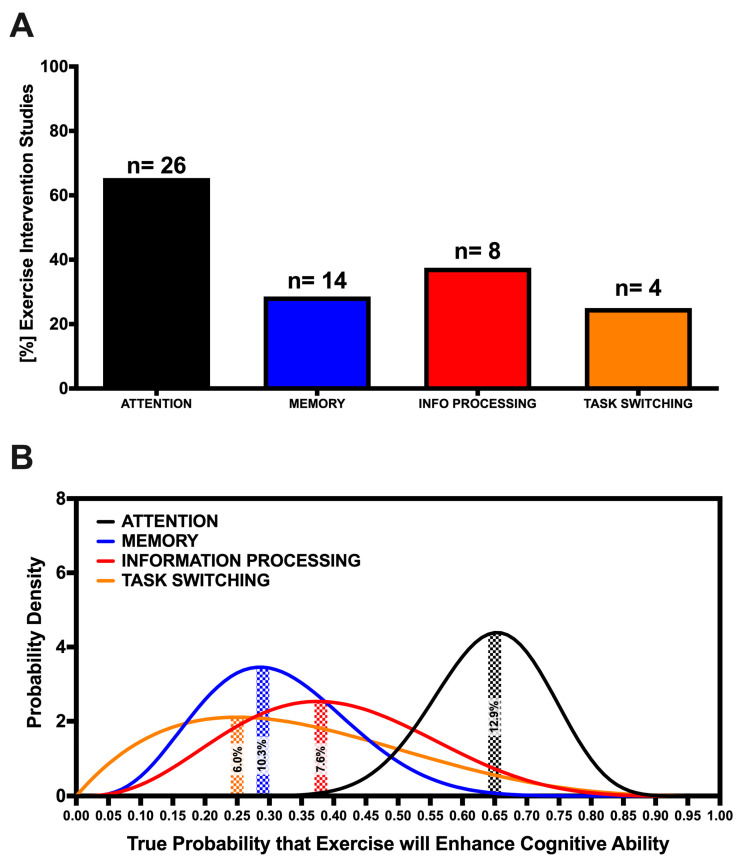
(**A**) Percent of included studies finding a positive effect of exercise on attention, memory, information processing and task-switching cognitive abilities. ‘*n*’ values represent the total number of included studies examining each cognitive ability. (**B**) Probability density functions for the true success rates of a study to find attention (*black trace*), memory (*blue*), information processing and task-switching (orange) performance improvements following exercise, respectively. Shaded areas represent the probability (/100%) that the observed success rate, given the findings of the included studies, reflects the true success rate.

**Figure 6:**
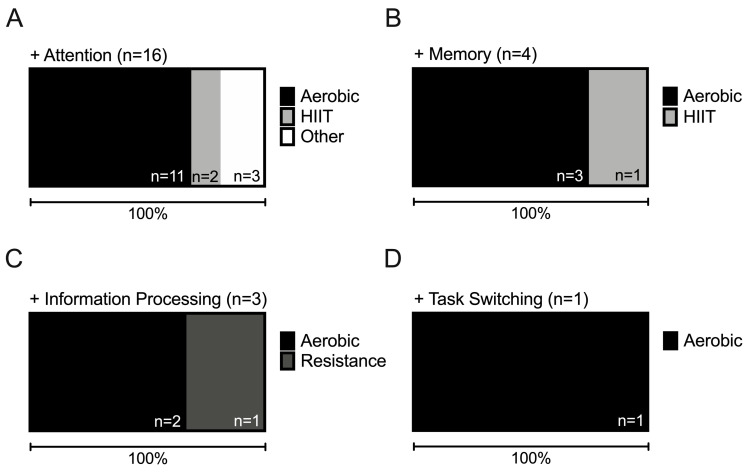
Contribution (in %) and number (*n*) of studies finding (**A**) Attention, (**B**) Memory, (**C**) Information Processing, and (**D**) Task-Switching improvement following exercise, separated by Aerobic, HIIT, Resistance and ‘Other’ exercise types.

Twenty-six studies identified in this review examined attentional skills before and after an exercise intervention among 1185 participants (Mean = 45.6, SD = 44.7), with a mean age of 22.89 years (SD = 2.74). These studies investigated how selective attention, sustained attention, visual, and spatial attention performance were affected by various types of exercise that lasted from 10 to 187 min. Researchers used the Stroop Test, Trail Making Test, and Sustained Attention to Response Task, among others (see Table 3), to assess attentional ability in the included studies. Overall, 17 studies (65.38% success rate) found exercise to improve attentional ability (Figure 5A). The confidence that this observed success rate represented the true success rate for an exercise intervention to improve attention given the number of included studies is 12.7% (Figure 5B). Twelve of the seventeen (70.59%) studies showing a positive effect of exercise on attention used aerobic exercise, two (12.50%) used high intensity interval training (HIIT), and three (18.75%) used alternate forms of exercise, including an exhaustive soldier training program, a mix of strength training, circuit training, indoor hockey, and Baduanjin (Figure 6A, Table 3). These studies found exercise to improve reaction time, sustained attention, visuospatial attention and attentional concentration. Moreover, three studies applied brain imaging techniques to examine the effect of exercise on attention. Ref. [129] and [140] both utilised EEG and showed larger P3 amplitudes in exercise group participants. Similarly, Ref. [127] used magnetic resonance imaging (MRI) and observed significantly lower grey matter cerebral blood flow (CBF) 10 min post-exercise compared to baseline and significantly lower global white matter CBF 10- and 40-min post-exercise. According to Laplace’s rule of succession, the probability that a new study would find exercise to improve attentional ability given the included findings is 64.29%.

#### 6.2.2. The Effect of Exercise on Memory

Fourteen intervention studies explored the effect of exercise on the memory abilities of 611 participants (Mean = 43.64, SD = 36.62) (Mean age = 22.96, SD = 2.71). Each study implemented a single exercise intervention session that varied from 10 to 60 min, (Mean = 29.29 min, SD = 12.84 min). Three studies used variations of the N-back task to investigate working memory while the remaining studies examined long- and short-term memory using the Free-Recall Memory Test, Paired Associations test and Vocabulary Learning task (Table 3). Four of the fourteen studies found exercise to improve memory performance (observed success rate of 28.57%; Figure 5A), with the remaining studies finding no significant effect (Table 3). The probability that the observed success rate here reflects the true success rate for exercise to improve memory ability is 10.3% (Figure 5B). Thirteen of the fourteen studies implemented an aerobic exercise intervention, ranging from low to high intensity. However, only three studies found a significant effect of exercise on memory performance in comparison to a control [142,150,154]. Alternatively, [157] showed that a single 20 min HIIT session was able to significantly improve memory performance on a Mnemonic-Similarity Task (Table 3). Overall, aerobic exercise and HIIT interventions contributed 75% and 25% of all positive effects on memory ability (Figure 6B). The probability of a future study demonstrating a positive effect of exercise on memory, according to Laplace’s rule of succession given the included data, is 31.25%.

#### 6.2.3. The Effect of Exercise on Information Processing

Eight studies assessed the effect of exercise on information processing in 489 (Mean = 61.13, SD = 41.51) participants, who were 23.17 (SD = 2.39) years of age. Three of the eight studies found exercise significantly improved information processing, resulting in an observed success rate of 37.5% (Figure 5A) with a 7.6% probability of this value reflecting the true success rate (Figure 5B). Each of the included studies were acute in nature and ranged from 10 to 35 min (Table 3). Seven of the eight included studies utilised aerobic exercise interventions, ranging from low to high intensity with two of these studies (28.57%) finding exercise to significantly improve information processing ability (Figure 6C). Specifically, [158] found that 15 min of moderate intensity jogging significantly improved TMT-A performance while [159] demonstrated that 30 min of jogging significantly improved performance on the Fitts’ Law task. The remaining study examined the effect of low, moderate and high intensity resistance exercise on information processing speed in comparison to a non-active control [160] (Table 3). Results showed that exercise significantly improved performance on the Paced Auditory Serial Addition Task (PASAT). According to Laplace’s rule of succession, there is a 40% probability of observing a positive effect of exercise on information processing ability in a future study, given the included study results presented here.

#### 6.2.4. The Effect of Exercise on Task-Switching

Finally, four studies assessed task-switching abilities in response to exercise among 362 participants (Mean = 90.5, SD = 47.89), who were aged 22.26 (SD = 1.57) years on average. Each of these studies implemented a single aerobic exercise session, ranging from 15 to 35 min (Table 3). Oberste, and colleagues [155] examined the effect of low, moderate and high intensity cycling, whereas [125] and [132] examined the effect of moderate intensity cycling, and [158] examined the effect of moderate intensity jogging. Each of these studies used the TMT-B or the Oral Trails B to assess task-switching ability. Only [132] found a significant effect of exercise on performance resulting in an observed success rate of 25% for exercise to enhance task-switching ability (Figure 5A). The probability of this success rate representing the true success rate given the included studies is 6.0% (Figure 5B). Finally, applying Laplace’s rule of succession, the probability that a new study would find exercise to improve task-switching ability, given the included studies, is 33.33%.

## 7. Discussion

Phase two of this dual systematic review aimed to examine the effect of physical exercise on the cognitive abilities identified to be important during AVG play, as highlighted in phase 1. Among the studies investigating each cognitive ability, participants showed significant improvements in attention, memory, information processing, and task-switching following exercise interventions in 65.38%, 28.57%, 37.50% and 25% of them, respectively (Figure 5A). Overall, these results suggest that despite the overwhelming positive effect of physical exercise on mood and physical health in young healthy adults, the effect of exercise on cognitive ability is less clear, with only attention appearing to show some benefit. However, it is important to note that none of the included studies demonstrated cognitive performance decrements following exercise. Moreover, we showed that the probability the observed success rates reflected the true success rates of exercise interventions to improve the different cognitive abilities was low (Figure 5B), suggesting that further research is required. Thirdly, we observe in our analyses that although the majority of studies investigated the effect of aerobic exercise on cognitive ability, alternate exercise types, such as resistance training and HIIT, show promise to improve cognitive ability and require further research. In light of this, we provide recommendations to guide future work in this area to establish the optimal parameters under which exercise can best enhance cognitive ability. Finally, we discuss the importance of exercise for AVG performance and how cognitive benefits that may be gained through exercise can benefit esports athletes looking to improve.

Our results indicate that the chance of finding an exercise intervention to improve cognitive ability is relatively low in healthy young adults, specifically for memory, information processing and task-switching abilities. While this finding appears to contradict previous reviews and meta analyses that have reported small to moderate positive effects of exercise on cognitive abilities [162,163,164,165,166], it is important to note that these reviews investigated specific populations such as older adults, children and clinical populations. Moreover, meta-analytical findings suggest that children and older adults can expect greater temporary cognitive benefits than other age groups following a single exercise session [166], which supports our findings that young healthy adults appear to confer only a small cognitive benefit from exercise. Previous reviews examining the effect of exercise on specific cognitive abilities in healthy young to middle aged adults indicate only a small positive effect of exercise on cognition (e.g., [46]). In support of these findings, the studies in our review did not find exercise to negatively affect cognitive ability, and despite the low observed success rates among studies included here, the combination of significant positive and null effects may corroborate the overall small positive effect of exercise on cognitive ability seen among these previous reviews. Taken together, there remains potential for exercise to improve cognitive ability in addition to its well-established effects on mood and physical health in young healthy adults (e.g., [167,168]), but further work is required to bolster or refute these initial studies. Importantly, we see that with a larger amount of evidence, exercise is more likely have a positive effect on attention, with 65.38% of included studies reporting significant improvements in performance. As the number of studies that examined the effect of exercise on attention was equal to the number that examined the effect of exercise on memory, information processing and task-switching combined, it may be that the benefit of exercise for these other cognitive abilities will manifest with further research as well.

Using Bayes’ Theorem to create probability density functions, we determined the probability that any of our observed success rates reflected the true success rate of exercise’s effect on cognition. This analysis demonstrated that due to the low number of studies investigating the effect of exercise on each cognitive ability, it remains very probable that an exercise intervention would improve information processing and task-switching abilities more than 50% of the time. From this insight, we acknowledge the importance of further work to increase confidence in the true effect of exercise on these cognitive abilities. Although additional research may exist that did not meet the inclusion criteria for our review, we caution the interpretation of research studies conducted without a control group, not conducted as a pre-post design and not examining specific cognitive abilities (i.e., examining executive functioning), as these cannot convincingly conclude on the true effect of exercise on specific cognitive abilities.

In this review, the majority of studies examined the effect of aerobic exercise on cognition (Figure 6). While support for the efficacy of aerobic exercise for enhancing cognitive ability is evident as previously reported [169,170,171]), we see evidence among the few studies included here that alternate exercise types, such as resistance exercise and HIIT, may also be efficacious for improving cognitive ability. For example, single (20 min) and multiple session (6 × 12–24 min) HIIT interventions were found to have significant benefits for performance on the Visuomotor skill acquisition task (VAT) and Attentional Network Test (ANT), respectively. These results corroborate suggestions that high-intensity interventions are more beneficial for complex attention than interventions of lower intensity [29,172]. Furthermore, other exercise types, such as coordinative exercise, are suggested to promise even more pronounced cognitive enhancements compared to aerobic exercise, resistance training and mixed exercise types [173]. While more work is needed to conclude on effects of resistance exercise, HIIT and other exercise types on cognitive ability, there is optimism that continued work may show these exercise types to be equally or more efficacious compared to aerobic exercise, especially when considering optimal dosage and intensity for young healthy adults.

### 7.1. Mechanistic Effects of Exercise and Video Game Play in the Brain

Previous work has demonstrated numerous mechanisms by which cognition is augmented by exercise and video gaming, respectively. For example, [174] and [175], respectively, found exercise and avg play to increase hippocampal volume, a structure implicated in numerous cognitive functions, including memory. Exercise has also been found to promote hippocampal neurogenesis [176] and globally, increase antioxidant capacity [177] and glycogen stores ([178] and [179]), decreasing risk of cognitive decline. Alternatively, gaming has been shown to induce regional increases in grey matter volume as well as improvements in neural plasticity and regional activity in both the Dorsolateral Prefrontal Cortex (DLPFC) [175] and fronto-parietal networks [180]. These areas are key for numerous cognitive attributes, including visual attention, memory, information processing and executive function abilities. Overall, evidence would suggest that exercise may induce global cortical effects, while engagement with video games may confer benefit to specific regions required for optimal performance of select cognitive functions. However, further work is required to determine both whether exercise-mediated global effects are attributable to all forms of exercise and the extent to which video game play mediates plastic regional changes that promote enhanced cognitive functioning.

### 7.2. Limitations and Recommendations for Future Research

Given the variance in exercise types studied and cognitive outcomes measured, it is difficult to conclude on the optimal exercise interventions for enhancing cognition in young healthy adults. Here, we provide several recommendations for the field that would bolster future research to establish the optimal parameters under which physical exercise can best enhance cognitive ability (Table 4). Specifically, we recommend that by establishing the optimal type, duration and intensity of exercise interventions for cognitive improvement, we will better understand the potency of exercise for the enhancement of cognitive ability in healthy young adults. These recommendations are in line with a recent meta-regression conducted by [172], which suggests that considering additional qualitative (exercise type) and quantitative (duration) moderators of exercise-induced cognitive benefits will maximise the efficacy of exercise interventions.

### 7.3. Exercise for Esports

The number of studies investigating the role of exercise training for enhancing cognitive ability is steadily growing, as exercise is easily accessible, cost-effective and promises additional well established physical and mood benefits (e.g., [181]). Typically, professional esport careers are short lived with only one-in-five careers lasting two years or longer [7,182]). Furthermore, the average retirement age of esport athletes is 25 [183] meaning that years of intense practice and training occur during a time when the brains of these individuals are still developing [184]. Given that esports appear to engage cognitive resources to a great extent, leading to players being labelled as ‘cognitive athletes’ [5], and that exercise appears to show the most promise for enhancing attention, it stands to reason that esport athletes may experience cognitive benefits from exercise. As recent meta-analytical (e.g., [164]) and experimental (e.g., [185] evidence suggests that younger individuals may be more likely to experience cognitive benefits from exercise, it may be in young aspiring gamers’ interests to allocate more time to physical exercise. In turn, this may give them a competitive advantage that may be the difference between a lucrative career as a professional esports athlete or not. In addition to the cognitive benefits outlined in this review, the well documented physical and mental health benefits of exercise suggest that it should be a staple in any elite esport training program.

## 8. Conclusions

This dual-systematic review has synthesised existing literature on the converging effect of gaming and exercise on cognitive ability. Group and intervention study results from Phase 1 demonstrate that attention, memory, information processing and task-switching abilities are cognitive abilities demanded by and likely important for success in AVGs. In light of these findings, Phase 2 examined the effect of exercise on these same identified cognitive skills. Our results demonstrate that aerobic exercise interventions are most effectively used to enhance attentional ability. Other exercise types, such as HIIT and resistance exercise, show promise but are understudied to date. Although recent reviews demonstrate that exercise has a low to moderate positive effect on cognition, our review highlights that a lack of research, particularly among young healthy adults, makes it difficult to make definitive conclusions. Furthermore, the lack of standardisation within the literature also hinders the strength of our conclusions. Therefore, we provide recommendations to guide future research to identify the optimal exercise types, durations and intensities to maximize the benefit of exercise on cognition. Finally, introducing exercise into the daily routine of professional esport athletes may not only provide physical and mental health benefits, but may confer cognitive benefits to give them an edge over fellow competitors.

## Figures and Tables

**Table 1:** Action video games (AVG) intervention study characteristics organised according to cognitive ability.

Cognitive Ability	Authors (Year)	Sample Size	Age: Mean (± SD)	AVG(s)	Dosage	Cognitive Test(s)	Result
Attention	Azizi et al. (2017) [54]	40	25.75 (± 3.95)	Call of Duty: Modern Warfare 2	10 × 1 h (2 weeks)	VST	=
	Boot et al. (2008) [55]	82	21.50 (± 2.30)	Medal of Honor	20 h	UFOV	=
	Feng, Spence, and Pratt (2007) [56]	20	N/A (± NA) †	Medal of Honor: Pacific Assault	10 h (over max 4 weeks)	UFOV	*
	Green and Bavelier (2003) [14]	16	NA (± NA) †	Medal of Honor: Allied Assault	10 × 1 h	UFOV, ABT and Enumeration Task.	*
	Hutchinson et al. (2016) [57]	45	NA (± NA) †	Call of Duty: Modern Warfare 3	10 × 1 h	Simon Task	*
	Li et al. (2016) [58]	16	23.50 (± NA)	Unreal Tournament 2004	10 h	Visuomotor Control Task	*
	Nelson and Strachan (2009) [59]	40	21.25 (± NA)	Unreal Tournament	1 h	Location Task	=
	Oei and Patterson (2015) [22]	59	21.78 (± 1.76)	Modern Combat, Metal Gear Solid, Super Sniper, Deer Hunter	20 h	ABT. Filter Task. VST. ADT	*
	Sanchez (2012) [60]	60	NA (± NA) †	Halo: Combat Evolved	25 min	Paper Folding and Card Rotations Tests	*
	Schlickum et al. (2009) [61]	30	NA (± NA) †	Half Life	Five weeks	Two VR Endoscopic Surgical Simulators	*
	Schubert et al. (2015) [62]	62	24.95 (± 3.76)	Medal of Honor	15 days	TVA test	=
Memory	Blacker et al. (2014) [63]	34	20.53 (± 2.57)	Call of Duty MW3 and Black Ops	30 h (over 30 days)	Change Detection Task and Colour Wheel	*
	Boot et al. (2008) [55]	82	21.50 (± 2.30)	Medal of Honor	20 h	Visual STM, Spatial Memory, CBT Tasks	=
	Sanchez (2012) [60]	60	NA (± NA) †	Halo: Combat Evolved	25 min	Automated Operation Span	=
	Schubert et al. (2015) [62]	62	24.95 (± 3.76)	Medal of Honor	15 days	TVA Task	=
Information Processing	Bailey and West (2013) [64]	31	22.13 (± 4.82)	Unreal Tournament 3	10 × 1 h	Emotion Search Task	=
	Boot et al. (2008) [55]	82	21.50 (± 2.30)	Medal of Honor	20 h	Mental Rotations Test	*
	Feng, Spence, and Pratt (2007) [56]	20	N/A (± NA) †	Medal of Honor: Pacific Assault	10 h (over max 4 weeks)	Mental Rotations Test	*
	Green and Bavelier (2006) [65]	32	21.15 (± NA)	Medal of Honor: Allied Assault	30 h	Enumeration Task	*
	Li et al. (2010) [66]	25	25.35 (± NA)	Unreal Tournament 4 and Call of Duty 2	50 h	Lateral Masking Paradigm	*
	Nelson and Strachan (2009) [59]	40	21.25 (± NA)	Unreal Tournament	1 h	Matching Figures Task	=
	Van Ravenzwaaij et al. (2014) [67]	45	20.00 (± 1.80)	Unreal Tournament 2004	20 h (5 sessions)	Moving Dots Task	=
Task-Switching	Chiappe et al. (2013) [68]	22	22.00 (± NA)	Ghost Recon, Advanced War Fighter 2, and Unreal Tournament 3	5 h/week (10 weeks)	MATB	*
	Green et al. (2012) [19]	35	25.70 (± 0.90)	Unreal Tournament 2004 and Call of Duty 2	50 h (average of 8.5 weeks)	Switch-Cost Paradigm	*
	Green and Bavelier (2006) [65]	32	21.15 (± NA)	Medal of Honor: Allied Assault	30 h	MOT Task	*
	Strobach et al. (2012) [69]	32	24.70 (± 3.76)	Medal of Honor	15 h	Dual-Task and TSP	*
	Wu and Spence (2013) [70]	60	NA (± NA) †	Medal of Honor: Pacific Assault	10 h	DST	*

* AVG group performed significantly better than the control. ^=^ No significant effect of AVG play. VST: Visual Search Task. ABT: Attentional Blink Task. UFOV: Useful Field of View. ADT: Auditory Detection Task. TVA: Theory of Visual Attention STM: Short-term Memory. CBT: Corsi Block-Tapping. MATB: Multi-Atrribute Task Battery. MOT: Multiple Object Tracking. DST: Dual Search Task. TSP: Task-Switching Paradigm † age data not available but used undergraduate students.

**Table 3:** Study characteristics of exercise intervention studies organised according to the cognitive skill assessed, Exercise Type and Intensity.

Cognitive Ability	Authors (Year)	Sample Size	Age: Mean (±SD)	Exercise Type	Dosage	Intensity	Cognitive Test	Results
Attention	Chandra et al. (2010) [125]	15	24.45 (± 1.35)	Aerobic Exercise (Ergometry cycling)	10 min	Low	SRT	*
	Douris et al. (2018) [126]	20	23.4 (± 2)	Aerobic Exercise (Ergometry Cycling)	30 min	Moderate	Stroop	*
	Kan et al. (2019) [127]	30	25.60 (± 4.9)	Aerobic Exercise (Cycling)	20 min	Low	SART	*
	Audiffren et al. (2008) [128]	17	22.00 (± 1.17)	Aerobic Exercise (Ergometry Cycling)	35 min	Moderate	Auditory two-CRT	*
	Chrismas et al. (2019) [129]	11	27.00 (± NA)	Aerobic Exercise (Treadmill walking)	30 min	Moderate	SRT, CRT	*
	Chang et al. (2015) [130]	30	20.92 (± 2.65)	Aerobic Exercise (cycling)	30 min	Moderate (70–85% HRmax)	ANT	*
	MacIntosh et al. (2014) [131]	16	26.70 (± 4.10)	Aerobic exercise (Cycling)	20 min	Moderate (70% HRmax)	SART	=
	Moreau et al. (2015) [40]	67	29.73 (± 7.83)	Aerobic exercise (treadmill, spinning bike, or rowing machine)	40 min	Moderate	Surface Development	*
	Murray and Russoniello (2012) [132]	120	20.86 (± 2.84)	Aerobic exercise (cycling)	30 min	Moderate (~75% HR max)	SRT, two-CRT and 4-CRT	*
	Pontifex et al. (2015) [133]	34	19.30 (± 0.90)	Aerobic Exercise (treadmill running)	20 min	Moderate (70% HRmax)	Three-stimulus Oddball task	*
	Reddy et al. (2014) [134]	42	19.60 (± NA)	Aerobic Exercise (cycling)	17 min	Moderate	Clinical Test of Reaction Time	=
	Nanda et al. 2013 [135]	10	19.50 (± 0.90)	Aerobic exercise (cycling)	30 min	Moderate (60–70% HRreserve)	Feature Match and Polygon task	=
	Sato et al. (2010) [136]	8	19.40 (± 1.00)	Aerobic Exercise (walking)	30 min	Moderate	PVT	*
	Thomas et al. (2016A) [137]	36	24.00 (± 3.00)	Aerobic exercise (Cycling)	17 min	Moderate	VAT	*
	Snow et al. (2016) [138]	16	25.70 (± 3.10)	Aerobic exercise (cycling)	30 min	Moderate (60% VO^2^peak)	Continuous Tracking Task	=
	Ashnager et al. (2015) [139]	29	23.00 (± 1.97)	Aerobic Exercise (Cycling)	30 min	Moderate-High	SART	*
	Basso et al. (2015) [140]	85	22.21 (± 0.45)	Aerobic Exercise (Ergometry Cycling)	50 min	High	Digit Span Test	=
	Du Rietz et al. (2019) [141]	29	21.50 (± 2.52)	Aerobic Exercise (cycling)	20 min	High	Cued Continuous Performance Task	=
	Lo Bue-Estes et al. (2008) [142]	17	20.60 (± 1.60)	Aerobic exercise (Treadmill running)	20 min	High	ANAM	=
	Moore et al. (2012) [143]	30	22.00 (± NA)	Aerobic exercise (cycling ergometer)	60 min	High (90% ventilatory threshold)	Visual Discrimination Test	=
	Llorens et al. (2015) [144]	30	24.00 (± 3.00)	HIT (cycling)	21.5 min	High	Spatial Attention Task	=
	Thomas et al. (2016 B) [145]	48	24.00 (± 2.50)	HIT (Cycling)	20 min	High (90% of peak power output Wmax)	VAT	*
	De Sousa et al. (2018) [146]	109	24.00 (± 4.79)	HIIT (Sprint interval training)	6 × 12–24 min	High	ANT	*
	Li et al. (2015B) [147]	206	20.78 (± 1.10)	Baduanjin Exercise	60 min	Low	Schulte Grid Test	*
	Thomas et al. (2017) [148]	40	25.30 (± 3.60)	Strength Training, Circuit Training or Indoor Hockey	47.5 min	High	Visuomotor Skill Acquisition Task	*
	Sipaviciene et al. (2012) [149]	90	19.50 (± NA)	Physical exercise (Soldier training program. 3 different workloads)	187 min	High	Attentional Concentration Test	*
Memory	Hötting et al. (2016) [150]	81	22.00 (± 2.36)	Aerobic exercise (Cycling)	30 min	Low (<57% HR max) and High (80% HRmax)	Vocabulary Learning	=
	Yamazaki et al. (2017) [151]	14	22.00 (± 0.60)	Aerobic exercise (Cycling)	10 min	Low (30% VO^2^peak)	Spatial Working Memory Task	*
	Yamazaki et al. (2018) [152]	30	21.80 (± 1.70)	Aerobic exercise (cycling ergometer)	10 min	Low to Moderate	N Back (0-back and 2-back) Tests	=
	Chrismas et al. (2019) [129]	11	27.00 (± NA)	Aerobic exercise (Treadmill walking)	30 min	Moderate	Serial-3 and Serial-7 Subtractions	=
	Coles and Tomporowski (2008) [153]	18	22.20 (± 1.60)	Aerobic exercise (Ergometry Cycling)	40 min	Moderate	Free Recall Memory Test	=
	Lambourne (2012) [154]	16	22.00 (± 0.88)	Aerobic exercise (Ergometry Cycling)	25 min	Moderate	RNG test	=
	Moreau et al. (2015) [40]	67	29.73 (± 7.83)	Aerobic exercise (treadmills, spinning bikes, or rowing machines)	40 min	Moderate	Immediate Free Recall, Backward Digit Span, and Letter-number Sequencing	=
	Nanda et al. (2013) [135]	10	19.50 (± 0.90)	Aerobic exercise (Cycling)	30 min	Moderate (60–70% HRmax)	Paired Associations Task	=
	Weng et al. (2015) [155]	26	25.23 (± 0.56)	Aerobic exercise (cycling)	30 min	Moderate	2-Back Test	*
	Oberste et al. (2016) [156]	121	23.81 (± 3.68)	Aerobic exercise (cycling)	35 min	Low (45–50%), Moderate (65–70%) and High (85–90% HRmax)	Free Recall Task	=
	Stroth et al. (2010) [157]	75	22.70 (± 5.70)	Aerobic exercise (Treadmill Running)	30 min	Moderate–High	2-Back Test	=
	Lo Bue-Estes et al. (2008) [142]	17	20.60 (± 1.60)	Aerobic exercise (Treadmill Running)	20 min	High	Spatial Memory, Code Substitution and Working Memory	*
	Moore et al. (2012) [143]	30	22.00 (± NA)	Aerobic exercise (cycling ergometer)	60 min	High (90% ventilatory threshold)	Vigilance Test	=
	Heisz et al. (2017) [158]	95	21.00 (± 3.00)	HIIT (Cycle ergometer)	20 min	High	Mnemonic Similarity Task	*
Information Processing	Legrand et al. (2018) [159]	101	20.76 (± 1.22)	Aerobic Exercise (Jogging)	15 min	Moderate	TMT-A	*
	Moreau et al. (2015) [40]	67	29.73 (± 7.83)	Aerobic exercise (treadmill, spinning bike, or rowing machine)	40 min	Moderate	Mental Rotations Test	=
	Smith et al. (2018) [160]	19	21.30 (± NA)	Aerobic Exercise (treadmill jogging)	30 min	Moderate	Fitts’ Law	*
	Chrismas et al. (2019) [129]	11	27.00 (± NA)	Aerobic Exercise (Treadmill walking)	30 min	Moderate	RVIP	=
	Basso et al. (2015) [140]	85	22.21 (± 0.45)	Aerobic Exercise (Ergometry Cycling)	50 min	High	TMT-A	=
	Lo Bue-Estes et al. (2008) [142]	17	20.60 (± 1.60)	Aerobic Exercise (Treadmill running)	20 min	High	ANAM	=
	Oberste et al. (2016) [156]	121	23.81 (± 3.68)	Aerobic exercise (cycling)	35 min	Low (45–50% HRmax) Moderate (65–70% HRmax) High (85–90% HRmax)	TMT-A	=
	Chang and Etnier (2009) [161]	68	25.95 (± 3.2)	Resistance Exercise (6 muscle groups)	2 × 10 reps	Low (40% 10-RM), Moderate (70% 10-RM) and High (100% 10-RM)	PASAT	*
Task-Switching	Douris et al. (2018) [126]	20	23.4 (± 2)	Aerobic Exercise (Ergometry Cycling)	30 min	Moderate	Stroop	*
	Oberste et al. (2016) [156]	121	23.81 (± 3.68)	Aerobic exercise (cycling)	35 min	Low (45–50%) moderate (65–70%) high (85–90%)	TMT-B	=
	Murray and Russoniello (2012) [132]	120	21.05 (± NA)	Aerobic exercise (cycling)	30 min	Moderate (~75% HRmax)	TMT-B	*
	Legrand et al. (2018) [159]	101	20.76 (± 1.22)	Aerobic exercise (Jogging)	15 min	Moderate	TMT-B	=

* Exercise significantly improved cognitive ability. ^=^ No significant effect of exercise. SRT: Simple Reaction Time. CRT: Choice Reaction Time. ANT: Attentional Network Test. SART: Sustained Attention to Response Test. PVT: Psychomotor Vigilance Test. VAT: Visuomotor skill acquisition task. ANAM: Automated Neuropsyochological Assessments Metric. RVIP: Rapid Visual Information Processing. TMT: Trail Making Test. RNG: Random Number Generation.

**Table 4:** Future research recommendations to optimize exercise intervention moderators.

Exercise Moderator	Recommendations
Exercise Type	Include various exercise types (Aerobic|HIIT|Resistance Training|Circuit Training|Yoga|Coordinative|Cross-fit|etc.)
	Precisely report exercise descriptions
Exercise Intensity	Include various exercise intensities (Low, Moderate, High)
	Precisely describe intensity criteria (HR max|VO^2^|Other Objective Measures)
	Precisely report intensity variations and/or progressions
Exercise Duration	Vary exercise dosage (10/20/30 min, etc.)
	Precisely report exercise duration
	Examine single and multiple session interventions

## Supplementary Materials

The following are available online at https://www.mdpi.com/2076-3425/10/11/859/s1. Supplementary file S1: PRISMA checklist; file S2: Gaming and Cognition Syntax; file S3: Gaming and Cognition PEDro scale; file S4: Exercise and Cognition Syntax; file S5: Exercise and Cognition PEDro scale.

## References

[1] Gough C. (2019). Number of Active Video Gamers Worldwide from 2014 to 2021. In Statista- The Statistics Portal. https://www.statista.com/statistics/748044/number-video-gamers-world/.

[2] Lenhart A., Kahne J., Middaugh E., Macgill R.A., Evans C., Vitak J. (2008). Teens, Video Games, and Civics: Teens’ Gaming Experiences Are Diverse and Include Significant Social Interaction and Civic Engagement. Pew Internet Am. Life Proj..

[3] Granic I., Lobel A., Engels R.C. (2014). The benefits of playing video games. Am. Psychol..

[4] Stewart S. (2019). Video Game Industry Silently Taking over Entertainment World. http://www.ejinsight.com/20191022-video-game-industry-silently-taking-over-entertainment-world/.

[5] Campbell J.M., Toth J.A., Moran P.A., Kowal M., Exton C. (2018). eSports: A new window on neurocognitive expertise?. Progress in Brain Research.

[6] Toth A.J., Kowal M., Campbell M. (2019). The Color-Word Stroop Task Does Not Differentiate Cognitive Inhibition Ability Among Esports Gamers of Varying Expertise. Front. Psychol..

[7] Smithies T.D., Toth A.J., Conroy E., Ramsbottom N., Kowal M., Campbell M.J. (2020). Life After Esports: A Grand Field Challenge. Front. Psychol..

[8] Villani D., Carissoli C., Triberti S., Marchetti A., Gilli G., Riva G. (2018). Videogames for Emotion Regulation: A Systematic Review. Games Heal. J..

[9] Poppelaars M., Lichtwarck-Aschoff A., Kleinjan M., Granic I. (2018). The impact of explicit mental health messages in video games on players’ motivation and affect. Comput. Hum. Behav..

[10] De Araujo T.B., Silveira F.R., Souza D.L.S., Strey Y.T.M., Flores C.D., Webster R.S. (2016). Impact of video game genre on surgical skills development: A feasibility study. J. Surg. Res..

[11] Gauthier L.V., Kane C., Borstad A., Strahl N., Uswatte G., Taub E., Morris D., Hall A., Arakelian M., Mark V. (2017). Video Game Rehabilitation for Outpatient Stroke (VIGoROUS): Protocol for a multi-center comparative effectiveness trial of in-home gamified constraint-induced movement therapy for rehabilitation of chronic upper extremity hemiparesis. BMC Neurol..

[12] Przybylski A.K., Weinstein N. (2019). Violent video game engagement is not associated with adolescents’ aggressive behaviour: Evidence from a registered report. R. Soc. Open Sci..

[13] Kühn S., Kugler D.T., Schmalen K., Weichenberger M., Witt C., Gallinat J. (2019). Does playing violent video games cause aggression? A longitudinal intervention study. Mol. Psychiatry.

[14] Green C.S., Bavelier D. (2003). Action video game modifies visual selective attention. Nat. Cell Biol..

[15] Statista Research Department (2016). Number of League of Legends Monthly Active Users (MAU) from 2011 to 2016. https://www.statista.com/statistics/317099/number-lol-registered-users-worldwide/#:~:text=This%20statistic%20illustrates%20the%20number,attracted%2036%20million%20viewers%20worldwide.

[16] Gough C. CS:GO MAU Worldwide 2019. https://www.statista.com/statistics/808922/csgo-users-number/.

[17] Gough C. DOTA 2 MAU Worldwide 2019. https://www.statista.com/statistics/607472/dota2-users-number/.

[18] Kowal M., Toth A.J., Exton C., Campbell M. (2018). Different cognitive abilities displayed by action video gamers and non-gamers. Comput. Hum. Behav..

[19] Green C., Bavelier D. (2012). Learning, Attentional Control, and Action Video Games. Curr. Biol..

[20] Uttal D.H., Meadow N.G., Tipton E., Hand L.L., Alden A.R., Warren C., Newcombe N.S. (2013). The malleability of spatial skills: A meta-analysis of training studies. Psychol. Bull..

[21] Sala G., Tatlidil K.S., Gobet F. (2018). Video game training does not enhance cognitive ability: A comprehensive meta-analytic investigation. Psychol. Bull..

[22] Oei A.C., Patterson M.D. (2015). Enhancing perceptual and attentional skills requires common demands between the action video games and transfer tasks. Front. Psychol..

[23] Kaser R. Hitting the Gym Makes eSports Athletes more Successful. https://thenextweb.com/gaming/2019/05/01/esports-athletes-gym-training/.

[24] Ayenigbara I.O. (2018). Gaming Disorder and Effects of Gaming on Health: An Overview. J. Addict. Med. Ther. Sci..

[25] Mentzoni R.A., Brunborg G.S., Molde H., Myrseth H., Skouverøe K.J.M., Hetland J., Pallesen S. (2011). Problematic Video Game Use: Estimated Prevalence and Associations with Mental and Physical Health. Cyberpsychology, Behav. Soc. Netw..

[26] Rudolf K., Grieben C., Achtzehn S., Froböse I. Stress im eSport–Ein Einblick in Training und Wettkampf. Proceedings of the eSpor-t Conference Professionalisierung einer Subkultur.

[27] Schültz M. Science Shows that eSports Professionals are Real Athletes. https://www.dw.com/en/science-shows-that-esports-professionals-are-real-athletes/a-19084993.

[28] Takahashi D. (2018). Overwatch League Commissioner Nate Nanzer: Esports Profits are Light at the End of the Tunnel. https://venturebeat.com/2018/09/29/overwatch-league-commissioner-nate-nanzer-esports-profits-are-light-at-the-end-of-the-tunnel/.

[29] Chang Y., Labban J., Gapin J., Etnier J. (2012). The effects of acute exercise on cognitive performance: A meta-analysis. Brain Res..

[30] Etnier J.L., Wideman L., Labban J.D., Piepmeier A.T., Pendleton D.M., Dvorak K.K., Becofsky K. (2016). The Effects of Acute Exercise on Memory and Brain-Derived Neurotrophic Factor (BDNF). J. Sport Exerc. Psychol..

[31] Winter B., Breitenstein C., Mooren F.C., Voelker K., Fobker M., Lechtermann A., Krueger K., Fromme A., Korsukewitz C., Floel A. (2007). High impact running improves learning. Neurobiol. Learn. Mem..

[32] Statton M.A., Encarnacion M., Celnik P., Bastian A.J. (2015). A Single Bout of Moderate Aerobic Exercise Improves Motor Skill Acquisition. PLoS ONE.

[33] Cheng S.-T. (2016). Cognitive Reserve and the Prevention of Dementia: The Role of Physical and Cognitive Activities. Curr. Psychiatry Rep..

[34] Labban J.D., Etnier J.L. (2011). Effects of acute exercise on long-term memory. Res. Q. Exerc. Sport.

[35] Hötting K., Röder B. (2013). Beneficial effects of physical exercise on neuroplasticity and cognition. Neurosci. Biobehav. Rev..

[36] Tomporowski P.D., Pesce C. (2019). Exercise, sports, and performance arts benefit cognition via a common process. Psychol. Bull..

[37] Pontifex M.B., Hillman C.H., Fernhall B., Thompson K.M., Valentini T.A. (2009). The Effect of Acute Aerobic and Resistance Exercise on Working Memory. Med. Sci. Sports Exerc..

[38] Schmitt A., Upadhyay N., Martin J.A., Rojas S., Strüder H.K., Boecker H. (2019). Modulation of Distinct Intrinsic Resting State Brain Networks by Acute Exercise Bouts of Differing Intensity. Brain Plast..

[39] Moreau D., Conway A.R. (2014). The case for an ecological approach to cognitive training. Trends Cogn. Sci..

[40] Moreau D., Morrison A.B., Conway A.R.A. (2015). An ecological approach to cognitive enhancement: Complex motor training. Acta Psychol..

[41] Bherer L., Erickson K.I., Liu-Ambrose T. (2013). A Review of the Effects of Physical Activity and Exercise on Cognitive and Brain Functions in Older Adults. J. Aging Res..

[42] Lees C., Hopkins J. (2013). Peer reviewed: Effect of aerobic exercise on cognition, academic achievement, and psychosocial function in children: A systematic review of randomized control trials. Prev. Chronic Dis..

[43] Bard C., Fleury M. (1978). Influence of Imposed Metabolic Fatigue on Visual Capacity Components. Percept. Mot. Ski..

[44] Fleury M., Bard C., Jobin J., Carriers L. (1981). Influence of Different Types of Physical Fatigue on a Visual Detection Task. Percept. Mot. Ski..

[45] Hopkins M.E., Davis F.C., Vantieghem M.R., Whalen P.J., Bucci D.J. (2012). Differential effects of acute and regular physical exercise on cognition and affect. Neuroscience.

[46] Loprinzi P.D., Frith E., Edwards M.K., Sng E., Ashpole N. (2017). The Effects of Exercise on Memory Function Among Young to Middle-Aged Adults: Systematic Review and Recommendations for Future Research. Am. J. Heal. Promot..

[47] Thompson J.J., Blair M.R., Henrey A.J. (2014). Over the Hill at 24: Persistent Age-Related Cognitive-Motor Decline in Reaction Times in an Ecologically Valid Video Game Task Begins in Early Adulthood. PLoS ONE.

[48] Moher D., Liberati A., Tetzlaff J., Altman D.G. (2009). Preferred reporting items for systematic reviews and meta-analyses: The PRISMA statement. Ann. Intern. Med..

[49] Wagner M.G. On the Scientific Relevance of eSports. Proceedings of the International Conference on Internet Computing.

[50] Bediou B., Adams D.M., Mayer R.E., Tipton E., Green C.S., Bavelier D. (2018). Meta-analysis of action video game impact on perceptual, attentional, and cognitive skills. Psychol. Bull..

[51] Sherrington C., Herbert R.D., Maher C.G., Moseley A.M. (2000). PEDro. A database of randomized trials and systematic reviews in physiotherapy. Man. Ther..

[52] Gong Y., Huang Z., Christensen E., Gluud C. (2007). Ursodeoxycholic Acid for Patients with Primary Biliary Cirrhosis: An Updated Systematic Review and Meta-Analysis of Randomized Clinical Trials Using Bayesian Approach as Sensitivity Analyses. Am. J. Gastroenterol..

[53] Laplace P.S., Truscott F.W., Emory F.L. (1951). A Philosophical Essay on Probabilities.

[54] Azizi E., Abel L., Stainer M.J. (2016). The influence of action video game playing on eye movement behaviour during visual search in abstract, in-game and natural scenes. Atten. Percept. Psychophys..

[55] Boot W.R., Kramer A.F., Simons D.J., Fabiani M., Gratton G. (2008). The effects of video game playing on attention, memory, and executive control. Acta Psychol..

[56] Feng J., Spence I., Pratt J. (2007). Playing an Action Video Game Reduces Gender Differences in Spatial Cognition. Psychol. Sci..

[57] Hutchinson C.V., Barrett D.J.K., Nitka A.W., Raynes K. (2015). Action video game training reduces the Simon Effect. Psychon. Bull. Rev..

[58] Li L., Chen R., Chen J. (2016). Playing Action Video Games Improves Visuomotor Control. Psychol. Sci..

[59] Nelson R., Strachan I. (2009). Action and Puzzle Video Games Prime Different Speed/Accuracy Tradeoffs. Perception.

[60] Sanchez C.A. (2012). Enhancing visuospatial performance through video game training to increase learning in visuospatial science domains. Psychon. Bull. Rev..

[61] Schlickum M.K., Hedman L., Enochsson L., Kjellin A., Felländer-Tsai L. (2009). Systematic Video Game Training in Surgical Novices Improves Performance in Virtual Reality Endoscopic Surgical Simulators: A Prospective Randomized Study. World J. Surg..

[62] Schubert T., Finke K., Redel P., Kluckow S., Müller H., Strobach T. (2015). Video game experience and its influence on visual attention parameters: An investigation using the framework of the Theory of Visual Attention (TVA). Acta Psychol..

[63] Blacker K.J., Curby K.M., Klobusicky E., Chein J.M. (2014). Effects of action video game training on visual working memory. J. Exp. Psychol. Hum. Percept. Perform..

[64] Bailey K., West R. (2013). The effects of an action video game on visual and affective information processing. Brain Res..

[65] Green C.S., Bavelier D. (2006). Effect of action video games on the spatial distribution of visuospatial attention. J. Exp. Psychol. Hum. Percept. Perform..

[66] Li R., Polat U., Scalzo F., Bavelier D. (2010). Reducing backward masking through action game training. J. Vis..

[67] Van Ravenzwaaij D., Boekel W., Forstmann B.U., Ratcliff R., Wagenmakers E.-J. (2014). Action video games do not improve the speed of information processing in simple perceptual tasks. J. Exp. Psychol. Gen..

[68] Chiappe D., Conger M., Liao J., Caldwell J.L., Vu K.-P.L. (2013). Improving multi-tasking ability through action videogames. Appl. Ergon..

[69] Strobach T., Frensch P.A., Schubert T. (2012). Video game practice optimizes executive control skills in dual-task and task switching situations. Acta Psychol..

[70] Wu S., Spence I. (2013). Playing shooter and driving videogames improves top-down guidance in visual search. Atten. Percept. Psychophys..

[71] Luck S.J., Vogel E.K. (1997). The capacity of visual working memory for features and conjunctions. Nat. Cell Biol..

[72] Berch D.B., Krikorian R., Huha E.M. (1998). The Corsi Block-Tapping Task: Methodological and Theoretical Considerations. Brain Cogn..

[73] Jaeggi S.M., Seewer R., Nirkko A.C., Eckstein D., Schroth G., Groner R., Gutbrod K. (2003). Does excessive memory load attenuate activation in the prefrontal cortex? Load-dependent processing in single and dual tasks: Functional magnetic resonance imaging study. NeuroImage.

[74] Antzaka A., Lallier M., Meyer S., Diard J., Carreiras M., Valdois S. (2017). Enhancing reading performance through action video games: The role of visual attention span. Sci. Rep..

[75] Bavelier D., Achtman R., Mani M., Föcker J. (2012). Neural bases of selective attention in action video game players. Vis. Res..

[76] Castel A.D., Pratt J., Drummond E. (2005). The effects of action video game experience on the time course of inhibition of return and the efficiency of visual search. Acta Psychol..

[77] Chisholm J.D., Kingstone A. (2015). Action video game players’ visual search advantage extends to biologically relevant stimuli. Acta Psychol..

[78] Chisholm J.D., Hickey C., Theeuwes J., Kingstone A. (2010). Reduced attentional capture in action video game players. Atten. Percept. Psychophys..

[79] Chisholm J.D., Kingstone A. (2012). Improved top-down control reduces oculomotor capture: The case of action video game players. Atten. Percept. Psychophys..

[80] Dye M.W.G., Bavelier D. (2010). Differential development of visual attention skills in school-age children. Vis. Res..

[81] Dye M.W.G., Green C., Bavelier D. (2009). The development of attention skills in action video game players. Neuropsychologia.

[82] Gorbet D.J., Sergio L.E. (2018). Move faster, think later: Women who play action video games have quicker visually-guided responses with later onset visuomotor-related brain activity. PLoS ONE.

[83] Green C.S., Bavelier D. (2006). Enumeration versus multiple object tracking: The case of action video game players. Cognition.

[84] Howard C.J., Wilding R., Guest D. (2016). Light Video Game Play is Associated with Enhanced Visual Processing of Rapid Serial Visual Presentation Targets. Perception.

[85] Hubert-Wallander B., Green C.S., Sugarman M., Bavelier D. (2011). Changes in search rate but not in the dynamics of exogenous attention in action videogame players. Atten. Percept. Psychophys..

[86] Krishnan L., Kang A., Sperling G., Srinivasan R. (2012). Neural Strategies for Selective Attention Distinguish Fast-Action Video Game Players. Brain Topogr..

[87] Mack D.J., Ilg U.J. (2014). The effects of video game play on the characteristics of saccadic eye movements. Vis. Res..

[88] Mack D.J., Wiesmann H., Ilg U.J. (2016). Video game players show higher performance but no difference in speed of attention shifts. Acta Psychol..

[89] Morin-Moncet O., Therrien-Blanchet J.-M., Ferland M.C., Théoret H., West G.L. (2016). Action Video Game Playing Is Reflected In Enhanced Visuomotor Performance and Increased Corticospinal Excitability. PLoS ONE.

[90] Richardson A.E., Collaer M.L. (2011). Virtual Navigation Performance: The Relationship to Field of View and Prior Video Gaming Experience. Percept. Mot. Ski..

[91] Sungur H., Boduroglu A. (2012). Action video game players form more detailed representation of objects. Acta Psychol..

[92] Unsworth N., Redick T.S., McMillan B.D., Hambrick D.Z., Kane M.J., Engle R.W. (2015). Is Playing Video Games Related to Cognitive Abilities?. Psychol. Sci..

[93] Wang P., Zhu X.-T., Liu H.-H., Zhang Y.-W., Hu Y., Li H.-J., Zuo X.-N. (2017). Age-Related Cognitive Effects of Videogame Playing Across the Adult Life span. Games Heal. J..

[94] West G.L., Drisdelle B.L., Konishi K., Jackson J., Jolicoeur P., Bohbot V.D. (2015). Habitual action video game playing is associated with caudate nucleus-dependent navigational strategies. Proc. R. Soc. B Biol. Sci..

[95] Wilms I.L., Petersen A., Vangkilde S. (2013). Intensive video gaming improves encoding speed to visual short-term memory in young male adults. Acta Psychol..

[96] Wong N.H.L., Chang D.H.F. (2018). Attentional advantages in video-game experts are not related to perceptual tendencies. Sci. Rep..

[97] Appelbaum L.G., Cain M.S., Darling E.F., Mitroff S.R. (2013). Action video game playing is associated with improved visual sensitivity, but not alterations in visual sensory memory. Atten. Percept. Psychophys..

[98] Cain M.S., Prinzmetal W., Shimamura A.P., Landau A.N. (2014). Improved control of exogenous attention in action video game players. Front. Psychol..

[99] Clark K., Fleck M.S., Mitroff S.R. (2011). Enhanced change detection performance reveals improved strategy use in avid action video game players. Acta Psychol..

[100] Green C.S., Bavelier D. (2007). Action-Video-Game Experience Alters the Spatial Resolution of Vision. Psychol. Sci..

[101] Latham A.J., Patston L.L.M., Tippett L.J. (2014). The precision of experienced action video-game players: Line bisection reveals reduced leftward response bias. Atten. Percept. Psychophys..

[102] Pavan A., Boyce M., Ghin F. (2016). Action Video Games Improve Direction Discrimination of Parafoveal Translational Global Motion but Not Reaction Times. Perception.

[103] Pohl C., Kunde W., Ganz T., Conzelmann A., Pauli P., Kiesel A. (2014). Gaming to see: Action video gaming is associated with enhanced processing of masked stimuli. Front. Psychol..

[104] Richlan F., Schubert J., Mayer R., Hutzler F., Kronbichler M. (2017). Action video gaming and the brain: fMRI effects without behavioral effects in visual and verbal cognitive tasks. Brain Behav..

[105] Steenbergen L., Sellaro R., Stock A.-K., Beste C., Colzato L. (2015). Action Video Gaming and Cognitive Control: Playing First Person Shooter Games Is Associated with Improved Action Cascading but Not Inhibition. PLoS ONE.

[106] West G.L., Stevens S.A., Pun C., Pratt J. (2008). Visuospatial experience modulates attentional capture: Evidence from action video game players. J. Vis..

[107] Blacker K.J., Curby K.M. (2013). Enhanced visual short-term memory in action video game players. Atten. Percept. Psychophys..

[108] Leite P.M.C., Kludt R., Vignola G., Ma W.J., Green C.S., Bavelier D. (2016). Technology consumption and cognitive control: Contrasting action video game experience with media multitasking. Atten. Percept. Psychophys..

[109] Cain M.S., Landau A.N., Shimamura A.P. (2012). Action video game experience reduces the cost of switching tasks. Atten. Percept. Psychophys..

[110] Donohue S.E., Woldorff M.G., Mitroff S.R. (2010). Video game players show more precise multisensory temporal processing abilities. Atten. Percept. Psychophys..

[111] Gaspar J.G., Neider M.B., Crowell J.A., Lutz A., Kaczmarski H., Kramer A.F. (2014). Are gamers better crossers? An examination of action video game experience and dual task effects in a simulated street crossing task. Hum. Factors J. Hum. Factors Ergon. Soc..

[112] Karle J.W., Watter S., Shedden J.M. (2010). Task switching in video game players: Benefits of selective attention but not resistance to proactive interference. Acta Psychol..

[113] Ball K.K., Beard B.L., Roenker D.L., Miller R.L., Griggs D.S. (1988). Age and visual search: Expanding the useful field of view. JOSA A.

[114] Nissen M.J., Bullemer P. (1987). Attentional requirements of learning: Evidence from performance measures. Cogn. Psychol..

[115] Robertson I.H., Manly T., Andrade J., Baddeley B.T., Yiend J. (1997). Oops!’: Performance correlates of everyday attentional failures in traumatic brain injured and normal subjects. Neuropsychologia.

[116] Latham A.J., Patston L.L.M., Westermann C., Kirk I.J., Tippett L.J. (2013). Earlier Visual N1 Latencies in Expert Video-Game Players: A Temporal Basis of Enhanced Visuospatial Performance?. PLoS ONE.

[117] Bonnechère B., Jansen B., Omelina Ľ., Jan S.V.S. (2016). The use of commercial video games in rehabilitation. Int. J. Rehabil. Res..

[118] Zeng N., Pope Z., Lee J.E., Gao Z. (2016). A systematic review of active video games on rehabilitative outcomes among older patients. J. Sport Heal. Sci..

[119] Raab M., Lobinger B., Hoffmann S., Pizzera A., Laborde S. (2015). Performance Psychology: Perception, Action, Cognition, and Emotion.

[120] Pedraza-Ramirez I., Musculus L., Raab M., Laborde S. (2020). Setting the scientific stage for esports psychology: A systematic review. Int. Rev. Sport Exerc. Psychol..

[121] Powers K.L., Brooks P.J., Aldrich N.J., Palladino M.A., Alfieri L. (2013). Effects of video-game play on information processing: A meta-analytic investigation. Psychon. Bull. Rev..

[122] Couyoumdjian A., Sdoia S., Tempesta D., Curcio G., Rastellini E., De Gennaro L., Ferrara M. (2010). The effects of sleep and sleep deprivation on task-switching performance. J. Sleep Res..

[123] Whitney P., Hinson J.M., Nusbaum A.T. (2019). A dynamic attentional control framework for understanding sleep deprivation effects on cognition. Prog. Brain Res..

[124] Hariohm K., Prakash V., Kumar S. (2015). Quantity and quality of randomized controlled trials published by Indian physiotherapists. Perspect. Clin. Res..

[125] Chandra A.M., Ghosh S., Barman S., Iqbal R., Sadhu N. (2010). Effect of Exercise and Heat-Load on Simple Reaction Time of University Students. Int. J. Occup. Saf. Ergon..

[126] Douris P.C., Handrakis J.P., Apergis D., Mangus R.B., Patel R., Limtao J., Platonova S., Gregorio A., Luty E. (2018). The Effects of Aerobic Exercise and Gaming on Cognitive Performance. J. Hum. Kinet..

[127] Kan B., Speelman C., Nosaka K. (2019). Cognitive demand of eccentric versus concentric cycling and its effects on post-exercise attention and vigilance. Graefe’s Arch. Clin. Exp. Ophthalmol..

[128] Audiffren M., Tomporowski P.D., Zagrodnik J. (2008). Acute aerobic exercise and information processing: Energizing motor processes during a choice reaction time task. Acta Psychol..

[129] Chrismas B.C.R., Taylor L., Cherif A., Sayegh S., Bailey D.P. (2019). Breaking up prolonged sitting with moderate-intensity walking improves attention and executive function in Qatari females. PLoS ONE.

[130] Chang Y.-K., Pesce C., Chiang Y.-T., Kuo C.-Y., Fong D.-Y. (2015). Antecedent acute cycling exercise affects attention control: An ERP study using attention network test. Front. Hum. Neurosci..

[131] MacIntosh B.J., Crane D.E., Sage M.D., Rajab A.S., Donahue M.J., McIlroy W.E., Middleton L.E. (2014). Impact of a Single Bout of Aerobic Exercise on Regional Brain Perfusion and Activation Responses in Healthy Young Adults. PLoS ONE.

[132] Murray N.P., Russoniello C. (2012). Acute Physical Activity on Cognitive Function: A Heart Rate Variability Examination. Appl. Psychophysiol. Biofeedback.

[133] Pontifex M.B., Parks A.C., Henning D.A., Kamijo K. (2014). Single bouts of exercise selectively sustain attentional processes. Psychophysiology.

[134] Reddy S., Eckner J., Kutcher J.S. (2014). Effect of Acute Exercise on Clinically Measured Reaction Time in Collegiate Athletes. Med. Sci. Sports Exerc..

[135] Nanda B., Manjunatha S. (2013). The Acute Effects of a Single Bout of Moderate-intensity Aerobic Exercise on Cognitive Functions in Healthy Adult Males. J. Clin. Diagn. Res..

[136] Sato T., Kubo T., Ebara T., Takeyama H., Inoue T., Iwanishi M., Tachi N., Itani T., Kamijima M. (2010). Brief hourly exercise during night work can help maintain workers’ performance. Ind. Heal..

[137] Thomas R., Beck M.M., Lind R.R., Johnsen L.K., Geertsen S.S., Christiansen L., Ritz C., Roig M., Lundbye-Jensen J. (2016). Acute Exercise and Motor Memory Consolidation: The Role of Exercise Timing. Neural Plast..

[138] Snow N.J., Mang C.S., Roig M., McDonnell M.N., Campbell K.L., Boyd L. (2016). The Effect of an Acute Bout of Moderate-Intensity Aerobic Exercise on Motor Learning of a Continuous Tracking Task. PLoS ONE.

[139] Ashnagar Z., Shadmehr A., Jalaei S. (2015). The effects of acute bout of cycling on auditory & visual reaction times. J. Bodyw. Mov. Ther..

[140] Basso J.C., Shang A., Elman M., Karmouta R., Suzuki W.A. (2015). Acute Exercise Improves Prefrontal Cortex but not Hippocampal Function in Healthy Adults. J. Int. Neuropsychol. Soc..

[141] Du Rietz E., Barker A.R., Michelini G., Rommel A.-S., Vainieri I., Asherson P., Kuntsi J. (2019). Beneficial effects of acute high-intensity exercise on electrophysiological indices of attention processes in young adult men. Behav. Brain Res..

[142] Bue-Estes C.L. (2008). Short-term exercise to exhaustion and its effects on cognitive function in young women. Percept. Mot. Ski..

[143] Moore R.D., Romine M.W., O’Connor P.J., Tomporowski P.D. (2012). The influence of exercise-induced fatigue on cognitive function. J. Sports Sci..

[144] Llorens F., Sanabria D., Huertas F. (2015). The Influence of Acute Intense Exercise on Exogenous Spatial Attention Depends on Physical Fitness Level. Exp. Psychol..

[145] Thomas R., Johnsen L.K., Geertsen S.S., Christiansen L., Ritz C., Roig M., Lundbye-Jensen J. (2016). Acute Exercise and Motor Memory Consolidation: The Role of Exercise Intensity. PLoS ONE.

[146] De Sousa A.F.M., Medeiros A.R., Benitez-Flores S., Del Rosso S., Stults-Kolehmainen M., Boullosa D. (2018). Improvements in Attention and Cardiac Autonomic Modulation After a 2-Weeks Sprint Interval Training Program: A Fidelity Approach. Front. Physiol..

[147] Li M., Fang Q., Li J., Zheng X., Tao J., Yan X., Lin Q., Lan X., Chen B., Zheng G. (2015). The Effect of Chinese Traditional Exercise-Baduanjin on Physical and Psychological Well-Being of College Students: A Randomized Controlled Trial. PLoS ONE.

[148] Thomas R., Flindtgaard M., Skriver K., Geertsen S.S., Christiansen L., Johnsen L.K., Busk D.V.P., Bojsen-Møller E., Madsen M.J., Ritz C. (2016). Acute exercise and motor memory consolidation: Does exercise type play a role?. Scand. J. Med. Sci. Sports.

[149] Sipaviciene S., Dumciene A., Ramanauskienė I., Skurvydas A. (2012). Effect of single physical load of different duration and intensity on cognitive function. Medicina.

[150] Hötting K., Schickert N., Kaiser J., Röder B., Schmidt-Kassow M. (2016). The Effects of Acute Physical Exercise on Memory, Peripheral BDNF, and Cortisol in Young Adults. Neural Plast..

[151] Yamazaki Y., Sato D., Yamashiro K., Tsubaki A., Yamaguchi Y., Takehara N., Maruyama A. (2017). Inter-individual differences in exercise-induced spatial working memory improvement: A near-infrared spectroscopy study. Oxygen Transport to Tissue XXXIX.

[152] Yamazaki Y., Sato D., Yamashiro K., Tsubaki A., Takehara N., Uetake Y., Nakano S., Maruyama A. (2018). Inter-individual differences in working memory improvement after acute mild and moderate aerobic exercise. PLoS ONE.

[153] Coles K., Tomporowski P.D. (2008). Effects of acute exercise on executive processing, short-term and long-term memory. J. Sports Sci..

[154] Lambourne K. (2012). The Effects of Acute Exercise on Temporal Generalization. Q. J. Exp. Psychol..

[155] Weng T.B., Pierce G.L., Darling W.G., Voss M.W. (2015). Differential Effects of Acute Exercise on Distinct Aspects of Executive Function. Med. Sci. Sports Exerc..

[156] Oberste M., Bloch W., Hübner S.T., Zimmer P. (2016). Do Reported Effects of Acute Aerobic Exercise on Subsequent Higher Cognitive Performances Remain if Tested against an Instructed Self-Myofascial Release Training Control Group? A Randomized Controlled Trial. PLoS ONE.

[157] Stroth S., Reinhardt R.K., Thöne J., Hille K., Schneider M., Härtel S., Weidemann W., Bös K., Spitzer M. (2010). Impact of aerobic exercise training on cognitive functions and affect associated to the COMT polymorphism in young adults. Neurobiol. Learn. Mem..

[158] Heisz J.J., Clark I.B., Bonin K., Paolucci E.M., Michalski B., Becker S., Fahnestock M. (2017). The Effects of Physical Exercise and Cognitive Training on Memory and Neurotrophic Factors. J. Cogn. Neurosci..

[159] Legrand F., Albinet C., Canivet A., Gierski F., Morrone I., Besche-Richard C. (2018). Brief aerobic exercise immediately enhances visual attentional control and perceptual speed. Testing the mediating role of feelings of energy. Acta Psychol..

[160] Smith D.L., Claytor R.P. (2018). An acute bout of aerobic exercise reduces movement time in a Fitts’ task. PLoS ONE.

[161] Chang Y.-K., Etnier J.L. (2009). Exploring the Dose-Response Relationship between Resistance Exercise Intensity and Cognitive Function. J. Sport Exerc. Psychol..

[162] Roig M., Nordbrandt S., Geertsen S.S., Nielsen J.B. (2013). The effects of cardiovascular exercise on human memory: A review with meta-analysis. Neurosci. Biobehav. Rev..

[163] Sanders L.M.J., Hortobágyi T., Gemert S.L.B.-V., Van Der Zee E.A., Van Heuvelen M.J.G. (2019). Dose-response relationship between exercise and cognitive function in older adults with and without cognitive impairment: A systematic review and meta-analysis. PLoS ONE.

[164] Xue Y., Yang Y., Huang T. (2019). Effects of chronic exercise interventions on executive function among children and adolescents: A systematic review with meta-analysis. Br. J. Sports Med..

[165] Northey J.M., Cherbuin N., Pumpa K.L., Smee D.J., Rattray B. (2017). Exercise interventions for cognitive function in adults older than 50: A systematic review with meta-analysis. Br. J. Sports Med..

[166] Ludyga S., Gerber M., Brand S., Holsboer-Trachsler E., Pühse U. (2016). Acute effects of moderate aerobic exercise on specific aspects of executive function in different age and fitness groups: A meta-analysis. Psychophysiology.

[167] Penedo F.J., Dahn J.R. (2005). Exercise and well-being: A review of mental and physical health benefits associated with physical activity. Curr. Opin. Psychiatry.

[168] Mikkelsen K., Stojanovska L., Polenakovic M., Bosevski M., Apostolopoulos V. (2017). Exercise and mental health. Maturitas.

[169] McMorris T., Hale B.J. (2012). Differential effects of differing intensities of acute exercise on speed and accuracy of cognition: A meta-analytical investigation. Brain Cogn..

[170] Cassilhas R.C., Viana V.A.R., Grassmann V., Dos Santos R.V.T., Santos R.F., Tufik S., De Mello M.T. (2007). The Impact of Resistance Exercise on the Cognitive Function of the Elderly. Med. Sci. Sports Exerc..

[171] Jeon Y.K., Ha C.H. (2017). The effect of exercise intensity on brain derived neurotrophic factor and memory in adolescents. Environ. Heal. Prev. Med..

[172] Ludyga S., Gerber M., Pühse U., Looser V.N., Kamijo K. (2020). Systematic review and meta-analysis investigating moderators of long-term effects of exercise on cognition in healthy individuals. Nat. Hum. Behav..

[173] Bhattacharya T.K., Pence B.D., Ossyra J.M., Gibbons T.E., Perez S., McCusker R.H., Kelley K.W., Johnson R.W., Woods J.A., Rhodes J.S. (2015). Exercise but not (−)-epigallocatechin-3-gallate or beta-alanine enhances physical fitness, brain plasticity, and behavioral performance in mice. Physiol. Behav..

[174] Kuhn S., Gleich T., Lorenz R.C., Lindenberger U., Gallinat J. (2014). Playing Super Mario induces structural brain plasticity: Gray matter changes resulting from training with a commercial video game. Mol. Psychiatry.

[175] Inoue K., Okamoto M., Shibato J., Lee M.C., Matsui T., Rakwal R., Soya H. (2015). Long-Term Mild, rather than Intense, Exercise Enhances Adult Hippocampal Neurogenesis and Greatly Changes the Transcriptomic Profile of the Hippocampus. PLoS ONE.

[176] Nokia M.S., Lensu S., Ahtiainen J.P., Johansson P.P., Koch L.G., Britton S.L., Kainulainen H. (2016). Physical exercise increases adult hippocampal neurogenesis in male rats provided it is aerobic and sustained. J. Physiol..

[177] Camiletti-Moirón D., Aparicio V.A., Aranda P., Radák Z. (2013). Does exercise reduce brain oxidative stress? A systematic review. Scand. J. Med. Sci. Sports.

[178] Brown A.M. (2004). Brain glycogen re-awakened. J. Neurochem..

[179] Matsui T., Ishikawa T., Ito H., Okamoto M., Inoue K., Lee M.-C., Fujikawa T., Ichitani Y., Kawanaka K., Soya H. (2012). Brain glycogen supercompensation following exhaustive exercise. J. Physiol..

[180] Nikolaidis A., Voss M.W., Elee H., Vo L.T.K., Kramer A.F. (2014). Parietal plasticity after training with a complex video game is associated with individual differences in improvements in an untrained working memory task. Front. Hum. Neurosci..

[181] O’Connor P.J., Herring M.P., Caravalho A. (2010). Mental Health Benefits of Strength Training in Adults. Am. J. Lifestyle Med..

[182] Ward M.R., Harmon A.D. (2019). ESport Superstars. J. Sports Econ..

[183] Tani S. (2018). Life after eSports: What Happens when Pro Gamers Hang up the Joystick?. https://asia.nikkei.com/Business/Business-trends/Life-after-esports-What-happens-when-pro-gamers-hang-up-the-joystick.

[184] Giedd J.N. (2004). Structural Magnetic Resonance Imaging of the Adolescent Brain. Ann. N. Y. Acad. Sci..

[185] Hogan C.L., Mata J., Carstensen L.L. (2013). Exercise holds immediate benefits for affect and cognition in younger and older adults. Psychol. Aging.

