# Supplementary material for: Converging Evidence Supporting the Cognitive Link between Exercise and Esport Performance: A Dual Systematic Review
Source: Brain Sci. 2020 Nov 15;10(11):859. doi: 10.3390/brainsci10110859 (PMC7696945; doi:10.3390/brainsci10110859)
Supplement: Supplementary file 1 [file brainsci-10-00859-s001.zip › Supplementary final/Supplementary file 2 Phase 1_Gaming and cognition Syntax.docx]

**Phase 1.**

The inclusion criteria for the review were formulated by NR, AT, and MC. The search for Phase 1 of this review was conducted by NR. Identified articles were extracted and exported into Endnote (Clarivate Analytics), except those found via Google Scholar, where only the first 200 references were extracted, as suggested by Bramer, Rethlesfen, Kleijnen, and Franco (2017). This meant that NR screened each of the identified articles based on the inclusion criteria. Unless NR was 100% confident that an article could be excluded, articles were kept for further analysis. In the case that NR was unsure about whether an article should be included or not, AT and MC were involved in checking each reference and a collective decision was made.

**Action Video Games and Cognition Search Syntax:**

*Combine one of the Action Video Gaming terms with one of the cognition terms, separated by the logical operator “AND”.*

- *Action Video Gaming:* “video gam*” “esport*” “computer gam*” “action video gam*” “MOBA” “FPS” “RTS” “electronic sport*”
- *Cognition*: (cogn*, cognitive ability, “recall”, executive function”, “mental”, “processing”, “reaction time”, “memory”, “perception”, “cognitive performance”, “attention”, “response inhibition”, “intelligence”, “cognitive load”, “cognitive processes”, “task-switching”, “dual-task”, “spatial cognition”, “Spatial awareness”, “useful field of view”).

Filters ran: ‘RCTs only’ where applicable

Gaming and cognition search terms for each database (PubMed, Medline, PsychINFO, Google Scholar)

Results found for each search combination were transported into the RIS file (compatible with EndNote ™ Software) and sent to researcher’s mail. Next, each file was downloaded into dedicated folder and added to the EndNote library for further screening procedures.

for PubMed inbuilt filters used:

- Article Type: **Randomized Controlled Trial**
- Year: 1999-present.
- Language: English

for PsychINFO via EBSCO inbuilt filters used:

- TI – Title; AB – Abstract, KW – Keyword
- **Linked Full Text**
- Publication Year: **1999+**
- **Peer Reviewed**
- **English**
- Age Group: **Young Adulthood (18-29), Thirties (30-39)**

for Medline inbuilt filters used:

• TI – Title; AB – Abstract, KW – Keyword

• Scholarly (Peer Reviewed) Journals;

• Linked Full Text;

• English Language;

• Age Related: Young Adult: 19-24 years, Adult: 19-44 years

• Date of Publication: 1999

Terms combined:

- “video gam*” AND “cogn*”
- “video gam*” AND “cognitive ability”
- “video gam*” AND “recall”
- “video gam*” AND executive function”
- “video gam*” AND “mental”
- “video gam*” AND “processing”
- “video gam*” AND “reaction time”
- “video gam*” AND “memory”
- “video gam*” AND “perception”
- “video gam*” AND “cognitive performance”
- “video gam*” AND “attention”
- “video gam*” AND “response inhibition”
- “video gam*” AND “intelligence”
- “video gam*” AND “cognitive load”
- “video gam*” AND “cognitive processes”
- “video gam*” AND “task-switching”
- “video gam*” AND “dual-task”
- “video gam*” AND “spatial cognition”
- “video gam*” AND “Spatial awareness”
- “video gam*” AND “useful field of view”
- “esport*” AND “cogn*”
- “esport*” AND “cognitive ability”
- “esport*” AND “recall”
- “esport*” AND executive function”
- “esport*” AND “mental”
- “esport*” AND “processing”
- “esport*” AND “reaction time”
- “esport*” AND “memory”
- “esport*” AND “perception”
- “esport*” AND “cognitive performance”
- “esport*” AND “attention”
- “esport*” AND “response inhibition”
- “esport*” AND “intelligence”
- “esport*” AND “cognitive load”
- “esport*” AND “cognitive processes”
- “esport*” AND “task-switching”
- “esport*” AND “dual-task”
- “esport*” AND “spatial cognition”
- “esport*” AND “Spatial awareness”
- “esport*” AND “useful field of view”
- “computer gam*” AND “cogn*”
- “computer gam*” AND “cognitive ability”
- “computer gam*” AND “recall”
- “computer gam*” AND executive function”
- “computer gam*” AND “mental”
- “computer gam*” AND “processing”
- “computer gam*” AND “reaction time”
- “computer gam*” AND “memory”
- “computer gam*” AND “perception”
- “computer gam*” AND “cognitive performance”
- “computer gam*” AND “attention”
- “computer gam*” AND “response inhibition”
- “computer gam*” AND “intelligence”
- “computer gam*” AND “cognitive load”
- “computer gam*” AND “cognitive processes”
- “computer gam*” AND “task-switching”
- “computer gam*” AND “dual-task”
- “computer gam*” AND “spatial cognition”
- “computer gam*” AND “Spatial awareness”
- “computer gam*” AND “useful field of view”
- “action video gam*” AND “cogn*”
- “action video gam*” AND “cognitive ability”
- “action video gam*” AND “recall”
- “action video gam*” AND executive function”
- “action video gam*” AND “mental”
- “action video gam*” AND “processing”
- “action video gam*” AND “reaction time”
- “action video gam*” AND “memory”
- “action video gam*” AND “perception”
- “action video gam*” AND “cognitive performance”
- “action video gam*” AND “attention”
- “action video gam*” AND “response inhibition”
- “action video gam*” AND “intelligence”
- “action video gam*” AND “cognitive load”
- “action video gam*” AND “cognitive processes”
- “action video gam*” AND “task-switching”
- “action video gam*” AND “dual-task”
- “action video gam*” AND “spatial cognition”
- “action video gam*” AND “Spatial awareness”
- “action video gam*” AND “useful field of view”
- “MOBA” AND “cogn*”
- “MOBA” AND“cognitive ability”
- “MOBA” AND “recall”
- “MOBA” AND “executive function”
- “MOBA” AND “mental”
- “MOBA” AND “processing”
- “MOBA” AND “reaction time”
- “MOBA” AND “memory”
- “MOBA” AND “perception”
- “MOBA” AND “cognitive performance”
- “MOBA” AND “attention”
- “MOBA” AND “response inhibition”
- “MOBA” AND “intelligence”
- “MOBA” AND “cognitive load”
- “MOBA” AND “cognitive processes”
- “MOBA” AND “task-switching”
- “MOBA” AND “dual-task”
- “MOBA” AND “spatial cognition”
- “MOBA” AND “Spatial awareness”
- “MOBA” AND “useful field of view”
- “FPS” AND “cogn*”
- “FPS” AND “cognitive ability”
- “FPS” AND “recall”
- “FPS” AND executive function”
- “FPS” AND “mental”
- “FPS” AND “processing”
- “FPS” AND “reaction time”
- “FPS” AND “memory”
- “FPS” AND “perception”
- “FPS” AND “cognitive performance”
- “FPS” AND “attention”
- “FPS” AND “response inhibition”
- “FPS” AND “intelligence”
- “FPS” AND “cognitive load”
- “FPS” AND “cognitive processes”
- “FPS” AND “task-switching”
- “FPS” AND “dual-task”
- “FPS” AND “spatial cognition”
- “FPS” AND “Spatial awareness”
- “FPS” AND “useful field of view”
- “RTS” AND “cogn*”
- “RTS” AND “cognitive ability”
- “RTS” AND “recall”
- “RTS” AND executive function”
- “RTS” AND “mental”
- “RTS” AND “processing”
- “RTS” AND “reaction time”
- “RTS” AND “memory”
- “RTS” AND “perception”
- “RTS” AND “cognitive performance”
- “RTS” AND “attention”
- “RTS” AND “response inhibition”
- “RTS” AND “intelligence”
- “RTS” AND “cognitive load”
- “RTS” AND “cognitive processes”
- “RTS” AND “task-switching”
- “RTS” AND “dual-task”
- “RTS” AND “spatial cognition”
- “RTS” AND “Spatial awareness”
- “RTS” AND “useful field of view”
- “electronic sport*” AND “cogn*”
- “electronic sport*” AND “cognitive ability”
- “electronic sport*” AND “recall”
- “electronic sport*” AND “executive function”
- “electronic sport*” AND “mental”
- “electronic sport*” AND “processing”
- “electronic sport*” AND “reaction time”
- “electronic sport*” AND “memory”
- “electronic sport*” AND “perception”
- “electronic sport*” AND “cognitive performance”
- “electronic sport*” AND “attention”
- “electronic sport*” AND “response inhibition”
- “electronic sport*” AND “intelligence”
- “electronic sport*” AND “cognitive load”
- “electronic sport*” AND “cognitive processes”
- “electronic sport*” AND “task-switching”
- “electronic sport*” AND “dual-task”
- “electronic sport*” AND “spatial cognition”
- “electronic sport*” AND “Spatial awareness”
- “electronic sport*” AND “useful field of view”
