# Supplementary material for: Converging Evidence Supporting the Cognitive Link between Exercise and Esport Performance: A Dual Systematic Review
Source: Brain Sci. 2020 Nov 15;10(11):859. doi: 10.3390/brainsci10110859 (PMC7696945; doi:10.3390/brainsci10110859)
Supplement: Supplementary file 1 [file brainsci-10-00859-s001.zip › Supplementary final/Supplementary file 3_Gaming and cognition PEDro scale_FINAL.docx]

| **Study Name** | **Year** | **Q1** | **Q2** | **Q3** | **Q4** | **Q5** | **Q6** | **Q7** | **Q8** | **Q9** | **Q10** | **Q11** | **Total** |
| --- | --- | --- | --- | --- | --- | --- | --- | --- | --- | --- | --- | --- | --- |
| Azizi et al [1] | 2017 | 1 | 1 | 1 | 1 | 1 |  | 0 | 1 |  | 1 | 1 | 8 |
| Bailey and West [2] | 2013 | 1 | 1 | 1 | 1 | 1 |  | 0 | 1 |  | 1 | 1 | 8 |
| Blacker et al [3] | 2014 | 1 | 1 | 1 | 1 | 1 |  | 0 | 1 |  | 1 | 1 | 8 |
| Boot et al [4] | 2008 | 1 | 1 | 1 | 1 | 1 |  | 0 | 1 |  | 1 | 1 | 8 |
| Chiappe et al [5] | 2012 | 1 | 1 | 1 | 1 | 1 |  | 0 | 1 |  | 1 | 1 | 8 |
| Feng, Spence, & Pratt [6] | 2007 | 1 | 1 | 1 | 1 | 1 |  | 0 | 1 |  | 1 | 1 | 8 |
| Green & Bavelier [7] | 2003 | 1 | 1 | 1 | 1 | 1 |  | 0 | 1 |  | 1 | 1 | 8 |
| Green & Bavelier [8] | 2006 | 1 | 1 | 1 | 1 | 1 |  | 0 | 1 |  | 1 | 1 | 8 |
| Green et al [9] | 2012 | 1 | 1 | 1 | 1 | 1 |  | 0 | 1 |  | 1 | 1 | 8 |
| Hutchinson et al [10] | 2015 | 1 | 1 | 1 | 1 | 1 |  | 0 | 1 |  | 1 | 1 | 8 |
| Li et al [11] | 2016 | 1 | 1 | 1 | 1 | 1 |  | 0 | 1 |  | 1 | 1 | 8 |
| Li et al [12] | 2010 | 1 | 1 | 1 | 1 | 1 |  | 0 | 1 |  | 1 | 1 | 8 |
| Nelson & Strachan [13] | 2009 | 1 | 1 | 1 | 1 | 1 |  | 0 | 1 |  | 1 | 1 | 8 |
| Oei & Patterson [14] | 2015 | 1 | 1 | 1 | 1 | 1 |  | 0 | 1 |  | 1 | 1 | 8 |
| Sanchez [15] | 2011 | 1 | 1 | 1 | 1 | 1 |  | 0 | 1 |  | 1 | 1 | 8 |
| Schlickum et al [16] | 2009 | 1 | 1 | 1 | 1 | 1 |  | 0 | 1 |  | 1 | 1 | 8 |
| Schubert et al 2015 [17] | 2015 | 1 | 1 | 1 | 1 | 1 |  | 0 | 1 |  | 1 | 1 | 8 |
| Strobach et al [18] | 2012 | 1 | 1 | 1 | 1 | 1 |  | 0 | 1 |  | 1 | 1 | 8 |
| Van Ravenzwaij et al [19] | 2014 | 1 | 1 | 1 | 1 | 1 |  | 0 | 1 |  | 1 | 1 | 8 |
| Wu & Spence [20] | 2013 | 1 | 1 | 1 | 1 | 1 |  | 0 | 1 |  | 1 | 1 | 8 |

References

1. Azizi, E.; Abel, L.; Stainer, M.J. The influence of action video game playing on eye movement behaviour during visual search in abstract, in-game and natural scenes. *Attention, Perception, Psychophys.* **2016**, *79*, 484–497.
2. Bailey, K., & West, R. (2013). The effects of an action video game on visual and affective information processing. *Brain research*, *1504*, 35-46.
3. Blacker, K.J.; Curby, K.M.; Klobusicky, E.; Chein, J.M. Effects of action video game training on visual working memory. *J. Exp. Psychol. Hum. Percept. Perform.* **2014**, *40*, 1992–2004.
4. Boot, W.R.; Kramer, A.F.; Simons, D.J.; Fabiani, M.; Gratton, G. The effects of video game playing on attention, memory, and executive control. *Acta Psychol.* **2008**, *129*, 387–398.
5. Chiappe, D.; Conger, M.; Liao, J.; Caldwell, J.L.; Vu, K.-P.L. Improving multi-tasking ability through action videogames. *Appl. Ergon.* **2013**, *44*, 278–284.
6. Feng, J.; Spence, I.; Pratt, J. Playing an Action Video Game Reduces Gender Differences in Spatial Cognition. *Psychol. Sci.* **2007**, *18*, 850–855.
7. Green, C.S.; Bavelier, D. Action video game modifies visual selective attention. *Nat. Cell Biol.* **2003**, *423*, 534–537.
8. Green, C. S., & Bavelier, D. (2006). Effect of action video games on the spatial distribution of visuospatial attention. *Journal of experimental psychology: Human perception and performance*, *32*(6), 1465.
9. Green, C.; Bavelier, D. Learning, Attentional Control, and Action Video Games. *Curr. Biol.* **2012**, *22*, R197–R206.
10. Hutchinson, C.V.; Barrett, D.J.K.; Nitka, A.W.; Raynes, K. Action video game training reduces the Simon Effect. *Psychon. Bull. Rev.* **2015**, *23*, 587–592.
11. Li, L.; Chen, R.; Chen, J. Playing Action Video Games Improves Visuomotor Control. *Psychol. Sci.* **2016**, *27*, 1092–1108.
12. Li, R.; Polat, U.; Scalzo, F.; Bavelier, D. Reducing backward masking through action game training. *J. Vis.* **2010**, *10*, 33.
13. Nelson, R.; Strachan, I. Action and Puzzle Video Games Prime Different Speed/Accuracy Tradeoffs. *Perception* **2009**, *38*, 1678–1687.
14. Oei, A.C.; Patterson, M.D. Enhancing perceptual and attentional skills requires common demands between the action video games and transfer tasks. *Front. Psychol.* **2015**, *6*, 113.
15. Sanchez, C.A. Enhancing visuospatial performance through video game training to increase learning in visuospatial science domains. *Psychon. Bull. Rev.* **2012**, *19*, 58–65.
16. Schlickum, M.K.; Hedman, L.; Enochsson, L.; Kjellin, A.; Felländer-Tsai, L. Systematic Video Game Training in Surgical Novices Improves Performance in Virtual Reality Endoscopic Surgical Simulators: A Prospective Randomized Study. *World J. Surg.* **2009**, *33*, 2360–2367.
17. Schubert, T.; Finke, K.; Redel, P.; Kluckow, S.; Müller, H.; Strobach, T. Video game experience and its influence on visual attention parameters: An investigation using the framework of the Theory of Visual Attention (TVA). *Acta Psychol.* **2015**, *157*, 200–214.
18. Strobach, T.; Frensch, P.A.; Schubert, T. Video game practice optimizes executive control skills in dual-task and task switching situations. *Acta Psychol.* **2012**, *140*, 13–24.
19. Van Ravenzwaaij, D.; Boekel, W.; Forstmann, B.U.; Ratcliff, R.; Wagenmakers, E.-J. Action video games do not improve the speed of information processing in simple perceptual tasks. *J. Exp. Psychol. Gen.* **2014**, *143*, 1794–1805.
20. Wu, S.; Spence, I. Playing shooter and driving videogames improves top-down guidance in visual search. *Attention, Perception, Psychophys.* **2013**, *75*, 673–686.
