# Supplementary material for: Converging Evidence Supporting the Cognitive Link between Exercise and Esport Performance: A Dual Systematic Review
Source: Brain Sci. 2020 Nov 15;10(11):859. doi: 10.3390/brainsci10110859 (PMC7696945; doi:10.3390/brainsci10110859)
Supplement: Supplementary file 1 [file brainsci-10-00859-s001.zip › Supplementary final/Supplementary file 4_Exercise and cognition Syntax.docx]

**Phase 2.**

The inclusion criteria for the review were formulated by NR, AT, and MC. For Phase 2 of the review, both NR and MK conducted searches of the databases. MK searched PsychINFO and Medline, and NR searched PubMed and Google Scholar. The same filters were used on each database to deal with All identified articles were extracted and exported into Endnote (Clarivate Analytics), except those found via Google Scholar, where only the first 200 references were extracted, as per the Bramer et al. (2017). Both authors worked alongside each other in order to ensure inter-rater reliability. This involved discussing a select sample of articles at the beginning of the process to ensure that both authors were on the same page and that the inclusion criteria were clear. In the case that one of the authors was unsure about one study they consulted the other. If both were still unsure, AT and MC served as arbitrators and a collective decision was made.

**Exercise and Cognition Search Syntax:**

*Combine one of the Exercise terms with one of the cognition terms (informed by the results of Phase 1), separated by the logical operator “AND”.*

- *Exercise*: “exercise”, “physical activity”, “physical activity intensity”, “exercise intensity”, “strength training”, “physical exercise”, “resistance training” , “aerobic exercise”, “HIIT”, “High intensity interval training”, “Motor task training”, “endurance training” “fitness”
- *Cognition*: (“attention” “visual selective attention” “sustained attention”, “reaction time”, “memory”, “working memory” “visuospatial cognition” “visual working memory” “visual short term memory” “contrast sensitivity” “executive control” “information processing”).

Exercise and Cognition Search Terms for each Database (PubMed, Medline, PsychINFO, Google Scholar)

Initially, the same procedure as in Phase 1: gaming and cognition. Following the same search procedure there has been 62036 titles for screening. Due to the enormous amount of articles derived from the initial search, decided to run the search once more with additional filters available in each database, corresponding with our inclusion criteria.

for PubMed inbuilt filters used:

- Article Type: **Randomized Controlled Trial**
- Language: **English**
- Publication Year: **1999+**

Results found for each search combination were transported into the RIS file (compatible with EndNote ™ Software) and sent to researcher’s mail. Next, each file was downloaded into dedicated folder and added to the EndNote library for further screening procedures.

for PsycINFO (via EBSCO) inbuilt filters used:

- TI – Title; AB – Abstract, KW – Keyword
- **Linked Full Text**
- Publication Year: **1999+**
- **Peer Reviewed**
- **English**
- Age Group: **Young Adulthood (18-29), Thirties (30-39)**

With keywords combined* in the following manner :

(TI "Exercise" OR AB "Exercise" OR KW "Exercise")

AND

(TI "attention" OR AB "attention" OR KW "attention") etc.

TI – Title; AB – Abstract, KW – Keyword

Results found for each search combination were transported into the RIS file (compatible with EndNote ™ Software) and sent to researcher’s mail. Next, each file was downloaded into dedicated folder and added to the EndNote library for further screening procedures.

For Medline (via EBSCO) inbuilt filters used:

- TI – Title; AB – Abstract, KW – Keyword
- **Scholarly (Peer Reviewed)** Journals;
- **Linked Full Text;**
- **English** Language;
- Age Related: **Young Adult: 19-24 years, Adult: 19-44 years**
- Date of Publication: **1999**

With keywords combined* in the following manner :

(TI "Exercise" OR AB "Exercise" OR KW "Exercise")

AND

(TI "attention" OR AB "attention" OR KW "attention") etc.

Results found for each search combination were transported into the RIS file (compatible with EndNote ™ Software) and sent to researcher’s mail. Next, each file was downloaded into dedicated folder and added to the EndNote library for further screening procedures.

*Keywords combinations:

- “exercise” AND “attention”
- “exercise” AND “visual selective attention”
- “exercise” AND “sustained attention”
- “exercise” AND “reaction time”
- “exercise” AND “memory”
- “exercise” AND “working memory”
- “exercise” AND “visuospatial cognition”
- “exercise” AND “visual working memory”
- “exercise” AND “visual short term memory”
- “exercise” AND “contrast sensitivity”
- “exercise” AND “executive control”
- “exercise” AND “information processing”
- “Physical activity intensity” AND “attention”
- “Physical activity intensity” AND “visual selective attention”
- “Physical activity intensity” AND “sustained attention”
- “Physical activity intensity” AND “reaction time”
- “Physical activity intensity” AND “memory”
- “Physical activity intensity” AND “working memory”
- “Physical activity intensity” AND “visuospatial cognition”
- “Physical activity intensity” AND “visual working memory”
- “Physical activity intensity” AND “visual short term memory”
- “Physical activity intensity” AND “contrast sensitivity”
- “Physical activity intensity” AND “executive control”
- “Physical activity intensity” AND “information processing”
- “strength training” AND “attention”
- “strength training” AND “visual selective attention”
- “strength training” AND “sustained attention”
- “strength training” AND “reaction time”
- “strength training” AND “memory”
- “strength training” AND “working memory”
- “strength training” AND “visuospatial cognition”
- “strength training” AND “visual working memory”
- “strength training” AND “contrast sensitivity”
- “strength training” AND “executive control”
- “strength training” AND “information processing”
- “physical activity” AND “visual attention”
- “physical activity” AND “spatial attention”
- “physical activity” AND “reaction time”
- “physical activity” AND “sustained attention”
- “physical activity” AND “memory”
- “physical activity” AND “working memory”
- “physical activity” AND “visuospatial cognition”
- “physical activity” AND “visual working memory”
- “physical activity” AND “visual short term memory”
- “physical activity” AND “contrast sensitivity ”
- “physical activity” AND “executive control”
- “physical activity” AND “information processing”
- “exercise intensity” AND “attention”
- “exercise intensity” AND “sustained attention”
- “exercise intensity” AND “visual attention”
- “exercise intensity” AND “spatial attention”
- “exercise intensity” AND “reaction time”
- “exercise intensity” AND “memory”
- “exercise intensity” AND “working memory”
- “exercise intensity” AND “visuospatial cognition”
- “exercise intensity” AND “visual working memory”
- “exercise intensity” AND “visual short term memory”
- “exercise intensity” AND “contrast sensitivity”
- “exercise intensity” AND “information processing”
- “physical exercise” AND “attention”
- “physical exercise” AND “sustained attention”
- “physical exercise” AND “visual selective attention”
- “physical exercise” AND “reaction time”
- “physical exercise” AND “memory”
- “physical exercise” AND “working memory”
- “physical exercise” AND “visuospatial cognition”
- “physical exercise” AND “visual working memory”
- “physical exercise” AND “visual short term memory”
- “physical exercise” AND “contrast sensitivity”
- “physical exercise” AND “executive control”
- “physical exercise” AND “information processing”
- “resistance training” AND “attention”
- “resistance training” AND “visual selective attention”
- “resistance training” AND “sustained attention”
- “resistance training” AND “reaction time”
- “resistance training” AND “memory”
- “resistance training” AND “visuospatial cognition”
- “resistance training” AND “working memory”
- “resistance training” AND “visual working memory”
- “resistance training” AND “visual short term memory”
- “resistance training” AND “contrast sensitivity”
- “resistance training” AND “executive function”
- “resistance training” AND “information processing”
- ”Aerobic exercise” AND “attention”
- ”Aerobic exercise” AND “visual selective attention”
- “aerobic exercise” AND “sustained attention”
- ”Aerobic exercise” AND “spatial attention”
- ”Aerobic exercise” AND “reaction time”
- ”Aerobic exercise” AND “memory”
- “Aerobic exercise” AND “visuospatial cognition”
- “Aerobic exercise” AND “working memory”
- “Aerobic exercise” AND “visual working memory”
- “Aerobic exercise” AND “visual short term memory”
- “Aerobic exercise” AND “contrast sensitivity”
- “Aerobic exercise” AND “information processing”
- “HIIT” AND “attention”
- “HIIT” AND “visual selective attention”
- “HIIT” AND “sustained attention”
- “HIIT” AND “reaction time”
- “HIIT” AND “memory”
- “HIIT” AND “working memory”
- “HIIT” AND “visuospatial cognition”
- “HIIT” AND “visual working memory”
- “HIIT” AND “visual short term memory”
- “HIIT” AND “contrast sensitivity ”
- “HIIT” AND “executive control”
- “HIIT” AND “information processing”
- “High intensity interval training” AND “attention”
- “High intensity interval training” AND “visual selective attention”
- “High intensity interval training” AND “spatial attention”
- “High intensity interval training” AND “reaction time”
- “High intensity interval training” AND “memory”
- “High intensity interval training” AND “working memory”
- “High intensity interval training” AND “visuospatial cognition”
- “High intensity interval training” AND “visual working memory”
- “High intensity interval training” AND “visual short term memory”
- “High intensity interval training” AND “contrast sensitivity”
- “High intensity interval training” AND “executive control”
- “High intensity interval training” AND “information processing”
- “Motor task training” AND “attention”
- “Motor task training” AND “visual selective attention”
- “Motor task training” AND “spatial attention”
- “Motor task training” AND “reaction time”
- “Motor task training” AND “memory”
- “Motor task training” AND “working memory”
- “Motor task training” AND “visual working memory”
- “Motor task training” AND “visuospatial cognition”
- “Motor task training” AND “visual short term memory”
- “Motor task training” AND “contrast sensitivity”
- “Motor task training” AND “executive control”
- “Motor task training” AND “information processing”
- “endurance training” AND “attention”
- “endurance training” AND “visual selective attention”
- “endurance training” AND “sustained attention”
- “enhanced cognition” AND “visuospatial cognition”
- “endurance training” AND “reaction time”
- “endurance training” AND “memory”
- “endurance training” AND “working memory”
- “endurance training” AND “visual working memory”
- “endurance training” AND “visual short term memory”
- “endurance training” AND “contrast sensitivity”
- “endurance training” AND “executive control”
- “endurance training” AND “information processing”
- “fitness” AND “attention”
- “fitness” AND “visual selective attention”
- “fitness” AND “sustained attention”
- “fitness” AND “visuospatial cognition”
- “fitness” AND “reaction time”
- “fitness” AND “memory”
- “fitness” AND “working memory”
- “fitness” AND “visual working memory”
- “fitness” AND “visual short term memory”
- “fitness” AND “contrast sensitivity”
- “fitness” executive control”
- “fitness” AND “”information processing”
