# Supplementary material for: Converging Evidence Supporting the Cognitive Link between Exercise and Esport Performance: A Dual Systematic Review
Source: Brain Sci. 2020 Nov 15;10(11):859. doi: 10.3390/brainsci10110859 (PMC7696945; doi:10.3390/brainsci10110859)
Supplement: Supplementary file 1 [file brainsci-10-00859-s001.zip › Supplementary final/Supplementary file 5_Exercise and cognition PEDro scale_FINAL.docx]

| **Study Name** | **Year** | **Q1** | **Q2** | **Q3** | **Q4** | **Q5** | **Q6** | **Q7** | **Q8** | **Q9** | **Q10** | **Q11** | **Total** |
| --- | --- | --- | --- | --- | --- | --- | --- | --- | --- | --- | --- | --- | --- |
| Ashnager et al [1] | 2015 | 1 | 1 | 1 | 1 | 1 |  | 0 | 1 |  | 1 | 1 | 8 |
| Audiffren et al [2] | 2008 | 1 | 1 | 1 | 1 | 1 |  | 0 | 1 |  | 1 | 1 | 8 |
| Basso et al [3] | 2015 | 1 | 1 | 1 | 1 | 1 |  | 0 | 1 |  | 1 | 1 | 8 |
| Chandra et al [4] | 2010 | 1 | 1 | 1 | 1 | 1 |  | 0 | 1 |  | 1 | 1 | 8 |
| Chang and Etnier [5] | 2009 | 1 | 1 | 1 | 1 | 1 |  | 0 | 1 |  | 1 | 1 | 8 |
| Chang et al [6] | 2015 | 1 | 1 | 1 | 1 | 1 |  | 0 | 1 |  | 1 | 1 | 8 |
| Chrismas et al [7] | 2019 | 1 | 1 | 1 | 1 | 1 |  | 0 | 1 |  | 1 | 1 | 8 |
| Coles and Tamporowski [8] | 2008 | 1 | 1 | 1 | 1 | 1 |  | 0 | 1 |  | 1 | 1 | 8 |
| de Sousa et al [9] | 2018 | 1 | 1 | 1 | 1 | 1 |  | 0 | 1 |  | 1 | 1 | 8 |
| Douris et al [10] | 2018 | 1 | 1 | 1 | 1 | 1 |  | 0 | 1 |  | 1 | 1 | 8 |
| Du Rietz et al [11] | 2019 | 1 | 1 | 1 | 1 | 1 |  | 0 | 1 |  | 1 | 1 | 8 |
| Heisz et al [12] | 2017 | 1 | 1 | 1 | 1 | 1 |  | 0 | 1 |  | 1 | 1 | 8 |
| Hötting et al [13] | 2016 | 1 | 1 | 1 | 1 | 1 |  | 0 | 1 |  | 1 | 1 | 8 |
| Kan et al [14] | 2019 | 1 | 1 | 1 | 1 | 1 |  | 0 | 1 |  | 1 | 1 | 8 |
| Lambourne [15] | 2012 | 1 | 1 | 1 | 1 | 1 |  | 0 | 1 |  | 1 | 1 | 8 |
| Legrand et al [16] | 2018 | 1 | 1 | 1 | 1 | 1 |  | 0 | 1 |  | 1 | 1 | 8 |
| Li et al [17] | 2015 | 1 | 1 | 1 | 1 | 1 |  | 0 | 1 |  | 1 | 1 | 8 |
| Llorens et al [18] | 2015 | 1 | 1 | 1 | 1 | 1 |  | 0 | 1 |  | 1 | 1 | 8 |
| Lo Bue-Estes et al [19] | 2008 | 1 | 1 | 1 | 1 | 1 |  | 0 | 1 |  | 1 | 1 | 8 |
| MacIntosh et al [20] | 2014 | 1 | 1 | 1 | 1 | 1 |  | 0 | 1 |  | 1 | 1 | 8 |
| Moore et al [21] | 2012 | 1 | 1 | 1 | 1 | 1 |  | 0 | 1 |  | 1 | 1 | 8 |
| Moreau et al [22] | 2015 | 1 | 1 | 1 | 1 | 1 |  | 0 | 1 |  | 1 | 1 | 8 |
| Murray et al [23] | 2012 | 1 | 1 | 1 | 1 | 1 |  | 0 | 1 |  | 1 | 1 | 8 |
| Nanda et al [24] | 2013 | 1 | 1 | 1 | 1 | 1 |  | 0 | 1 |  | 1 | 1 | 8 |
| Oberste et al [25] | 2016 | 1 | 1 | 1 | 1 | 1 |  | 0 | 1 |  | 1 | 1 | 8 |
| Pontifex et al [26] | 2015 | 1 | 1 | 1 | 1 | 1 |  | 0 | 1 |  | 1 | 1 | 8 |
| Reddy et al [27] | 2014 | 1 | 1 | 1 | 1 | 1 |  | 0 | 1 |  | 1 | 1 | 8 |
| Sato et al [28] | 2010 | 1 | 1 | 1 | 1 | 1 |  | 0 | 1 |  | 1 | 1 | 8 |
| Sipaviciene et al [29] | 2012 | 1 | 1 | 1 | 1 | 1 |  | 0 | 1 |  | 1 | 1 | 8 |
| Smith et al [30] | 2018 | 1 | 1 | 1 | 1 | 1 |  | 0 | 1 |  | 1 | 1 | 8 |
| Thomas et al [31] | 2017 | 1 | 1 | 1 | 1 | 1 |  | 0 | 1 |  | 1 | 1 | 8 |
| Thomas et al [32] | 2016 | 1 | 1 | 1 | 1 | 1 |  | 0 | 1 |  | 1 | 1 | 8 |
| Thomas et al [33] | 2016 | 1 | 1 | 1 | 1 | 1 |  | 0 | 1 |  | 1 | 1 | 8 |
| Weng et al [34] | 2015 | 1 | 1 | 1 | 1 | 1 |  | 0 | 1 |  | 1 | 1 | 8 |
| Yamazaki et al [35] | 2018 | 1 | 1 | 1 | 1 | 1 |  | 0 | 1 |  | 1 | 1 | 8 |
| Yamazaki et al [36] | 2017 | 1 | 1 | 1 | 1 | 1 |  | 0 | 1 |  | 1 | 1 | 8 |

References

1. Ashnagar, Z.; Shadmehr, A.; Jalaei, S. The effects of acute bout of cycling on auditory & visual reaction times. *J. Bodyw. Mov. Ther.* **2015**, *19*, 268–272.
2. Audiffren, M.; Tomporowski, P.D.; Zagrodnik, J. Acute aerobic exercise and information processing: Energizing motor processes during a choice reaction time task. *Acta Psychol.* **2008**, *129*, 410–419.
3. Basso, J.C.; Shang, A.; Elman, M.; Karmouta, R.; Suzuki, W.A. Acute Exercise Improves Prefrontal Cortex but not Hippocampal Function in Healthy Adults. *J. Int. Neuropsychol. Soc.* **2015**, *21*, 791–801.
4. Chandra, A.M.; Ghosh, S.; Barman, S.; Iqbal, R.; Sadhu, N. Effect of Exercise and Heat-Load on Simple Reaction Time of University Students. *Int. J. Occup. Saf. Ergon.* **2010**, *16*, 497–505.
5. Chang, Y.-K.; Etnier, J.L. Exploring the Dose-Response Relationship between Resistance Exercise Intensity and Cognitive Function. *J. Sport Exerc. Psychol.* **2009**, *31*, 640–656.
6. Chang, Y.-K.; Pesce, C.; Chiang, Y.-T.; Kuo, C.-Y.; Fong, D.-Y. Antecedent acute cycling exercise affects attention control: An ERP study using attention network test. *Front. Hum. Neurosci.* **2015**, *9*, 156.
7. Chrismas, B.C.R.; Taylor, L.; Cherif, A.; Sayegh, S.; Bailey, D.P. Breaking up prolonged sitting with moderate-intensity walking improves attention and executive function in Qatari females. *PLoS ONE* **2019**, *14*, e0219565.
8. Coles, K.; Tomporowski, P.D. Effects of acute exercise on executive processing, short-term and long-term memory. *J. Sports Sci.* **2008**, *26*, 333–344.
9. De Sousa, A.F.M.; Medeiros, A.R.; Benitez-Flores, S.; Del Rosso, S.; Stults-Kolehmainen, M.; Boullosa, D. Improvements in Attention and Cardiac Autonomic Modulation After a 2-Weeks Sprint Interval Training Program: A Fidelity Approach. *Front. Physiol.* **2018**, *9*, doi:10.3389/fphys.2018.00241.
10. Douris, P.C.; Handrakis, J.P.; Apergis, D.; Mangus, R.B.; Patel, R.; Limtao, J.; Platonova, S.; Gregorio, A.; Luty, E. The Effects of Aerobic Exercise and Gaming on Cognitive Performance. *J. Hum. Kinet.* **2018**, *61*, 73–83.
11. Du Rietz, E.; Barker, A.R.; Michelini, G.; Rommel, A.-S.; Vainieri, I.; Asherson, P.; Kuntsi, J. Beneficial effects of acute high-intensity exercise on electrophysiological indices of attention processes in young adult men. *Behav. Brain Res.* **2019**, *359*, 474–484.
12. Heisz, J.J.; Clark, I.B.; Bonin, K.; Paolucci, E.M.; Michalski, B.; Becker, S.; Fahnestock, M. The Effects of Physical Exercise and Cognitive Training on Memory and Neurotrophic Factors. *J. Cogn. Neurosci.* **2017**, *29*, 1895–1907.
13. Hötting, K.; Schickert, N.; Kaiser, J.; Röder, B.; Schmidt-Kassow, M. The Effects of Acute Physical Exercise on Memory, Peripheral BDNF, and Cortisol in Young Adults. *Neural Plast.* **2016**, *2016*, 1–12.
14. Kan, B.; Speelman, C.; Nosaka, K. Cognitive demand of eccentric versus concentric cycling and its effects on post-exercise attention and vigilance. *Graefe’s Arch. Clin. Exp. Ophthalmol.* **2019**, *119*, 1599–1610.
15. Lambourne, K. The Effects of Acute Exercise on Temporal Generalization. *Q. J. Exp. Psychol.* **2012**, *65*, 526–540.
16. Legrand, F.; Albinet, C.; Canivet, A.; Gierski, F.; Morrone, I.; Besche-Richard, C. Brief aerobic exercise immediately enhances visual attentional control and perceptual speed. Testing the mediating role of feelings of energy. *Acta Psychol.* **2018**, *191*, 25–31.
17. Li, M.; Fang, Q.; Li, J.; Zheng, X.; Tao, J.; Yan, X.; Lin, Q.; Lan, X.; Chen, B.; Zheng, G.; et al. The Effect of Chinese Traditional Exercise-Baduanjin on Physical and Psychological Well-Being of College Students: A Randomized Controlled Trial. *PLoS ONE* **2015**, *10*, e0130544.
18. Llorens, F.; Sanabria, D.; Huertas, F. The Influence of Acute Intense Exercise on Exogenous Spatial Attention Depends on Physical Fitness Level. *Exp. Psychol.* **2015**, *62*, 20–29.
19. Bue-Estes, C.L. Short-term exercise to exhaustion and its effects on cognitive function in young women. *Percept. Mot. Ski.* **2008**, *107*, 933.
20. MacIntosh, B.J.; Crane, D.E.; Sage, M.D.; Rajab, A.S.; Donahue, M.J.; McIlroy, W.E.; Middleton, L.E. Impact of a Single Bout of Aerobic Exercise on Regional Brain Perfusion and Activation Responses in Healthy Young Adults. *PLoS ONE* **2014**, *9*, e85163.
21. Moore, R.D.; Romine, M.W.; O’Connor, P.J.; Tomporowski, P.D. The influence of exercise-induced fatigue on cognitive function. *J. Sports Sci.* **2012**, *30*, 841–850.
22. Basso, J.C.; Shang, A.; Elman, M.; Karmouta, R.; Suzuki, W.A. Acute Exercise Improves Prefrontal Cortex but not Hippocampal Function in Healthy Adults. *J. Int. Neuropsychol. Soc.* **2015**, *21*, 791–801.
23. Murray, N.P.; Russoniello, C. Acute Physical Activity on Cognitive Function: A Heart Rate Variability Examination. *Appl. Psychophysiol. Biofeedback* **2012**, *37*, 219–227.
24. Nanda., B., & Manjunatha, S. The Acute Effects of a Single Bout of Moderate-intensity Aerobic Exercise on Cognitive Functions in Healthy Adult Males. *J. Clin. Diagn. Res.* **2013**, *7*, 1883–1885.
25. Oberste, M.; Bloch, W.; Hübner, S.T.; Zimmer, P. Do Reported Effects of Acute Aerobic Exercise on Subsequent Higher Cognitive Performances Remain if Tested against an Instructed Self-Myofascial Release Training Control Group? A Randomized Controlled Trial. *PLoS ONE* **2016**, *11*, e0167818.
26. Pontifex, M.B.; Parks, A.C.; Henning, D.A.; Kamijo, K. Single bouts of exercise selectively sustain attentional processes. *Psychophysiology* **2014**, *52*, 618–25.
27. Reddy, S.; Eckner, J.; Kutcher, J.S. Effect of Acute Exercise on Clinically Measured Reaction Time in Collegiate Athletes. *Med. Sci. Sports Exerc.* **2014**, *46*, 429–434.
28. Sato, T.; Kubo, T.; Ebara, T.; Takeyama, H.; Inoue, T.; Iwanishi, M.; Tachi, N.; Itani, T.; Kamijima, M. Brief hourly exercise during night work can help maintain workers’ performance. *Ind. Heal.* **2010**, *48*, 470–477.
29. Sipaviciene, S.; Dumciene, A.; Ramanauskienė, I.; Skurvydas, A. Effect of single physical load of different duration and intensity on cognitive function. *Medicina* **2012**, *48*, 31.
30. Smith, D.L.; Claytor, R.P. An acute bout of aerobic exercise reduces movement time in a Fitts’ task. *PLoS ONE* **2018**, *13*, e0210195.
31. Thomas, R.; Flindtgaard, M.; Skriver, K.; Geertsen, S.S.; Christiansen, L.; Johnsen, L.K.; Busk, D.V.P.; Bojsen-Møller, E.; Madsen, M.J.; Ritz, C.; et al. Acute exercise and motor memory consolidation: Does exercise type play a role? *Scand. J. Med. Sci. Sports* **2016**, *27*, 1523–1532.
32. Thomas, R.; Johnsen, L.K.; Geertsen, S.S.; Christiansen, L.; Ritz, C.; Roig, M.; Lundbye-Jensen, J. Acute Exercise and Motor Memory Consolidation: The Role of Exercise Intensity. *PLoS ONE* **2016**, *11*, e0159589.
33. Thomas, R.; Beck, M.M.; Lind, R.R.; Johnsen, L.K.; Geertsen, S.S.; Christiansen, L.; Ritz, C.; Roig, M.; Lundbye-Jensen, J. Acute Exercise and Motor Memory Consolidation: The Role of Exercise Timing. *Neural Plast.* **2016**, *2016*, 1–11.
34. Weng, T.B.; Pierce, G.L.; Darling, W.G.; Voss, M.W. Differential Effects of Acute Exercise on Distinct Aspects of Executive Function. *Med. Sci. Sports Exerc.* **2015**, *47*, 1460–1469.
35. Yamazaki, Y.; Sato, D.; Yamashiro, K.; Tsubaki, A.; Takehara, N.; Uetake, Y.; Nakano, S.; Maruyama, A. Inter-individual differences in working memory improvement after acute mild and moderate aerobic exercise. *PLoS ONE* **2018**, *13*, e0210053.
36. Yamazaki, Y.; Sato, D.; Yamashiro, K.; Tsubaki, A.; Yamaguchi, Y.; Takehara, N.; Maruyama, A. Inter-individual differences in exercise-induced spatial working memory improvement: A near-infrared spectroscopy study. In *Oxygen Transport to Tissue XXXIX.*; Springer: Cham, Switzerland, 2017; pp. 81–88.
